# Mechanisms of Manganese(II) Oxidation by Filamentous Ascomycete Fungi Vary With Species and Time as a Function of Secretome Composition

**DOI:** 10.3389/fmicb.2021.610497

**Published:** 2021-02-10

**Authors:** Carolyn A. Zeiner, Samuel O. Purvine, Erika Zink, Si Wu, Ljiljana Paša-Tolić, Dominique L. Chaput, Cara M. Santelli, Colleen M. Hansel

**Affiliations:** ^1^Department of Biology, University of St. Thomas, Saint Paul, MN, United States; ^2^Environmental Molecular Sciences Laboratory, Pacific Northwest National Laboratory, Richland, WA, United States; ^3^Biological Sciences Laboratory, Pacific Northwest National Laboratory, Richland, WA, United States; ^4^Department of Chemistry and Biochemistry, The University of Oklahoma, Norman, OK, United States; ^5^Biosciences, Geoffrey Pope Building, University of Exeter, Exeter, United Kingdom; ^6^Department of Earth and Environmental Sciences, University of Minnesota, Minneapolis, MN, United States; ^7^Department of Marine Chemistry & Geochemistry, Woods Hole Oceanographic Institution, Woods Hole, MA, United States

**Keywords:** manganese oxides, filamentous fungi, geomicrobiology, proteomics, biomineralization, secretome

## Abstract

Manganese (Mn) oxides are among the strongest oxidants and sorbents in the environment, and Mn(II) oxidation to Mn(III/IV) (hydr)oxides includes both abiotic and microbially-mediated processes. While white-rot Basidiomycete fungi oxidize Mn(II) using laccases and manganese peroxidases in association with lignocellulose degradation, the mechanisms by which filamentous Ascomycete fungi oxidize Mn(II) and a physiological role for Mn(II) oxidation in these organisms remain poorly understood. Here we use a combination of chemical and in-gel assays and bulk mass spectrometry to demonstrate secretome-based Mn(II) oxidation in three phylogenetically diverse Ascomycetes that is mechanistically distinct from hyphal-associated Mn(II) oxidation on solid substrates. We show that Mn(II) oxidative capacity of these fungi is dictated by species-specific secreted enzymes and varies with secretome age, and we reveal the presence of both Cu-based and FAD-based Mn(II) oxidation mechanisms in all 3 species, demonstrating mechanistic redundancy. Specifically, we identify candidate Mn(II)-oxidizing enzymes as tyrosinase and glyoxal oxidase in *Stagonospora* sp. SRC1lsM3a, bilirubin oxidase in *Stagonospora* sp. and *Paraconiothyrium sporulosum* AP3s5-JAC2a, and GMC oxidoreductase in all 3 species, including *Pyrenochaeta* sp. DS3sAY3a. The diversity of the candidate Mn(II)-oxidizing enzymes identified in this study suggests that the ability of fungal secretomes to oxidize Mn(II) may be more widespread than previously thought.

## Introduction

Manganese (Mn) (III/IV) (hydr)oxide minerals are ubiquitous in the environment, including terrestrial and aquatic systems. Due to their small particle size, large surface area, and high sorptive and oxidative capacities, Mn oxides are among the most reactive mineral phases in the environment. Mn oxides can impact a variety of biogeochemical processes, including degradation of recalcitrant organic compounds such as humic acids (Stone and Morgan, 1984; Sunda and Kieber, 1994) and organic contaminants (Rubert and Pedersen, 2006), adsorption and redox cycling of trace metals (Nelson et al., 1999; Murray and Tebo, 2007), remediation of metal-contaminated waters (Santelli et al., 2010; Luan et al., 2012), and anaerobic respiration coupled to carbon oxidation (Nealson and Saffarini, 1994). Furthermore, Mn(II) oxidation has been implicated in degradation of lignocellulose (Glenn et al., 1986; Höfer and Schlosser, 1999), and Mn redox cycling in the soil has been shown to drive long-term litter decomposition rates in terrestrial ecosystems (Keiluweit et al., 2015; Jones et al., 2018), thereby playing a significant role in greenhouse gas (CO_2_) emissions and regulation of the global carbon (C) cycle. Due to the importance of Mn oxides in contaminant remediation and breakdown of recalcitrant C sources, isolation of Mn(II)-oxidizing microorganisms and elucidation of the underlying mechanisms has the potential to aid in large-scale environmental preservation efforts.

The mechanisms of Mn(II) oxidation to Mn(III/IV) (hydr)oxides include both abiotic and microbially-mediated processes. Abiotic oxidation of Mn(II) by molecular oxygen is thermodynamically prohibited at circumneutral pH, owing to an energetic barrier in the first electron transfer step from Mn(II) to Mn(III) (Luther, 2010). Complexation of Mn(II) to destabilizing ligands, mineral surfaces, and/or enzyme active sites removes this energetic barrier, allowing for rapid O_2_-induced Mn(II) oxidation to Mn(III), which may then be further oxidized or disproportionate to Mn(II) and Mn(IV) to ultimately precipitate Mn oxides (Bargar et al., 2005; Duckworth and Sposito, 2005; Madden and Hochella, 2005; Learman et al., 2011b). Furthermore, the reactive oxygen species (ROS) superoxide (O_2_^•–^) of biogenic or abiogenic origin rapidly oxidizes Mn(II) to Mn(III) under a wide range of conditions (Nico et al., 2002; Hansard et al., 2011; Learman et al., 2013). In the environment, precipitation of Mn(III/IV) oxide minerals is mediated to a great extent by either direct or indirect microbiological activity. A large and diverse group of Mn(II)-oxidizing bacteria (Tebo et al., 2004), fungi (Miyata et al., 2007), and algae (Chaput et al., 2019) have been identified to date, and research on the underlying mechanisms has begun to elucidate the roles of key enzymes and reactive metabolites.

Bacterial Mn(II) oxidation has been studied extensively in model organisms such as *Bacillus* sp. strain SG-1 (Bargar et al., 2005), *Pseudomonas putida* strains GB-1 (Brouwers et al., 1999) and MnB1 (Villalobos et al., 2003), and *Leptothrix discophora* strain SS-1 (Adams and Ghiorse, 1987). These organisms enzymatically oxidize Mn(II) using multicopper oxidases (MCOs) localized in an extracellular, exopolymeric matrix [reviewed in Tebo et al. (2004), Butterfield et al. (2013)]. Other studies have implicated extracellular heme peroxidases as Mn(II)-oxidizing enzymes in three alphaproteobacteria (Anderson et al., 2009; Andeer et al., 2015). For one of these organisms, *Roseobacter* sp. Azwk-3b, it has been shown that the heme peroxidase produces the ROS superoxide that directly oxidizes Mn(II) to Mn(III) (Learman et al., 2011a; Andeer et al., 2015). Similarly, an enzymatic superoxide-based Mn(II) oxidation mechanism has been recently identified in a cyanobacterium and several algal phototrophs (Chaput et al., 2019). While a physiological role of Mn(II) oxidation in bacteria remains largely enigmatic, energy conservation coupled to Mn(II) oxidation to Mn(III/IV) oxides has been suggested in several bacterial species and consortia (Ehrlich, 1983; Ehrlich and Salerno, 1990; Daye et al., 2019; Yu and Leadbetter, 2020).

Investigations of fungal Mn(II) oxidation have traditionally focused on model white-rot Basidiomycetes such as *Phanerochaete chrysosporium* due to their ability to effectively degrade lignocellulose from plant material. In these organisms, Mn(II) oxidation is directly linked to lignocellulose degradation and is catalyzed by extracellular enzymes including laccases (Höfer and Schlosser, 1999), Mn peroxidases (Glenn et al., 1986; Wariishi et al., 1992; Hofrichter, 2002), a cooperative combination of the two (Schlosser and Höfer, 2002), or related enzymes including dye-decolorizing peroxidases (Fernandez-Fueyo et al., 2015). These mechanisms are intimately linked to the cycling of ROS, as laccases can indirectly produce ROS as by products of Mn(II) or other substrate oxidation (Schlosser and Höfer, 2002), and Mn peroxidases require H_2_O_2_ as an electron acceptor (Wariishi et al., 1992). Both enzymes oxidize Mn(II) to Mn(III) complexes, which are either reduced back to Mn(II) coupled to lignocellulose oxidation, or abiotically disproportionate to form Mn(III/IV) oxides (Perez and Jeffries, 1992).

Significantly less is known about the mechanisms by which Mn(II) is oxidized by filamentous Ascomycete fungi, a ubiquitous and cosmopolitan yet understudied group. Recent work, however, has begun to elucidate these processes and distinguish them from those catalyzed by Basidiomycetes. Initial demonstration of an enzymatic Mn(II) oxidation mechanism distinct from Basidiomycete Mn peroxidases (de la Torre and Gomez-Alarcon, 1994) has been followed by several studies implicating secreted laccase-like MCOs (LMCOs). Mn(II) oxidation by LMCOs has been observed in several phylogenetically diverse Ascomycetes, demonstrating oxidation of traditional laccase substrates and inhibition by copper chelators (Miyata et al., 2004; Miyata et al., 2006a, b), and even suggesting a link between enzymatic Mn(II) oxidation and plant pathogenicity (Thompson et al., 2006). Interestingly, Ascomycete laccases and LMCOs (e.g., bilirubin oxidase, ascorbate oxidase) are phylogenetically distinct from each other and from Basidiomycete laccases (Hoegger et al., 2006), suggesting that different mechanisms may exist for each class of secreted enzyme. Indeed, in *Acremonium* sp. strain KR21-2, the Mn(II)-oxidizing LMCO has been recently identified as bilirubin oxidase rather than a true laccase (Tojo et al., 2020). Additionally, the physiological role of Ascomycete Mn(II) oxidation remains poorly understood. Unlike for Basidiomycetes, Mn(II) oxidation by Ascomycetes has not been linked to lignocellulose degradation or acquisition of other carbon or nutrient sources, although several Mn(II)-oxidizing Ascomycetes have demonstrated cellulose oxidation capacity (Nilsson et al., 1989; Shary et al., 2007), including the 3 species in this study (unpublished data, CM Santelli and CA Zeiner).

Previous work has demonstrated the involvement of both hyphal-associated reactive metabolites and secreted organic polymers in Mn(II) oxidation by filamentous Ascomycetes. Superoxide produced by transmembrane NADPH oxidases has been shown as the oxidant of Mn(II) to Mn(III) in three genera (*Stilbella*, *Stagonospora*, and *Pyrenochaeta*) during growth on agar-solidified medium (Hansel et al., 2012; Tang et al., 2013), demonstrating an interesting homology to the O_2_^•–^-mediated mechanism in the bacterium *R*. Azwk-3b (Learman et al., 2011a) and in microbial phototrophs (Chaput et al., 2019). Additionally, Mn(III/IV) oxide precipitation at distances away from the hyphae combined with observations of thin, carbonaceous filaments intimately associated with the mycogenic Mn oxides suggest a role for secreted proteins as a Mn(III) chelator and/or mineral nucleation template (Santelli et al., 2011; Tang et al., 2013). The ability of proteins secreted by these organisms to directly oxidize Mn(II), however, remains unknown.

In this study, we investigated extracellular Mn(II)-oxidizing proteins in the cell-free secretome of three phylogenetically diverse, Mn(II)-oxidizing, filamentous Ascomycete fungi: *Stagonospora* sp. SRC1lsM3a, *Pyrenochaeta* sp. DS3sAY3a, and *Paraconiothyrium sporulosum* AP3s5-JAC2a. We tested two main hypotheses:

1. Hypothesis 1: Mn(II) oxidation is directly catalyzed by extracellular proteins in the fungal secretome.
2. Hypothesis 2: Extracellular Mn(II)-oxidizing proteins vary by species and over time as a function of secretome composition.

By combining chemical and enzyme activity assays, gel electrophoresis, and bulk mass spectrometry, we demonstrated secretome-based Mn(II) oxidative capacity in each fungus that exhibited species-specific temporal patterns. We also identified a diverse suite of candidate Mn(II)-oxidizing enzymes in each organism.

## Materials and Methods

### Secretome Preparation and Initial Characterization

#### Fungal Species and Culture Medium

We investigated three Mn(II)-oxidizing Ascomycete fungi isolated from two locations. Two species were isolated from passive coal mine drainage treatment systems in central Pennsylvania that attenuate high concentrations of Mn (Santelli et al., 2010): *Stagonospora* sp. SRC1lsM3a and *Pyrenochaeta* sp. DS3sAY3a. The third species was isolated from Ashumet Pond, Massachusetts, a natural freshwater lake (Santelli et al., 2014): *Paraconiothyrium sporulosum* AP3s5-JAC2a. This field site was historically polluted with elevated concentrations of phosphate and metals, including Fe and Mn. The genomes of all 3 species are available on GenBank [accession numbers LXTA00000000 (*Stagonospora* sp.), LXSZ00000000 (*Pyrenochaeta* sp.), and LXPO00000000 (*P. sporulosum*)], and detailed analyses of their secretome composition have been published previously (Zeiner et al., 2016, 2017).

All fungal species were grown in HEPES-buffered (20 mM, pH 7) acetate-yeast extract (AY) medium (Miyata et al., 2004; Tang et al., 2013) supplemented with MnCl_2_ (0–200 μM). Fungal cultures were maintained on petri dishes containing agar-solidified (2% agar) AY medium with 200 μM Mn(II) (hereafter AY + Mn).

#### Culture Conditions and Secretome Harvesting

To prepare cell-free secretomes, fungi were grown in 100 mL liquid cultures in AY + Mn medium using 100 μL of blender-homogenized inocula. Preparation of the inoculum is described in the Supplementary Material.

Cultures were incubated at 21°C without agitation for 7, 14, or 21 days. For each fungus at each of the 3 time points, 5 individual 100 mL cultures were combined into 500 mL samples to maximize protein recovery. These 500 mL samples were prepared in quadruplicate. Upon harvesting, bulk biomass was removed and discarded, and the spent medium was filtered through a 0.45 μm polyethersulfone membrane (VWR) to remove remaining cells and Mn oxides. Samples were then concentrated using a centrifugal filter with a 10 kDa, low protein adhesion membrane (EMD Millipore) according to the manufacturer’s instructions (see Supplementary Material). These concentrated ∼250 μL secretome samples were then stored at −80°C until analysis.

#### Protein Quantification

Protein in secretome samples was quantified using a Pierce^TM^ BCA protein assay kit (Thermo Fisher Scientific) as conducted previously (Smith et al., 1985). The quantity of protein recovered from 500 mL secretome samples generally ranged between 250 and 1,000 μg, depending on species and secretome age, with protein quantity increasing over time.

#### Mn Oxide Quantification

The amount of Mn(III/IV) oxides generated by the secretome samples was quantified with a colorimetric method using the Leucoberbelin blue (LBB) reagent (Krumbein and Altmann, 1973). LBB produces a deep blue color in the presence of Mn in any oxidation state higher than Mn(II). Samples were incubated with LBB in the dark for 15 min, followed by measurement of their spectrophotometic absorbance at 620 nm on a SpectraMax i3 or Tecan Infinite MPlex microplate reader. Standard curves were prepared with LBB and KMnO_4_.

### Proteinaceous Nature of Mn(II)-Oxidizing Factors

To determine whether Mn(II) oxidation is catalyzed by extracellular proteins in the secretomes, we boiled the samples (to denature proteins) or treated them with a protease prior to Mn oxide quantification. Samples were boiled at 100°C for 30 min or were incubated with 1 mg mL^–1^ proteinase K (fungal, Sigma) at 20°C for 2 h. Mn(II) was then added in excess (in the range of 100–250 μM depending on the sample). Samples were incubated with Mn(II) for 1 h followed by Mn(III/IV) oxide quantification via LBB.

To distinguish potential enzymatic Mn(II) oxidation in the cell-free secretome from previously established superoxide-mediated Mn(II) oxidation via hyphal-associated NADPH oxidases in these species (Tang et al., 2013), concentrated secretome samples were incubated with Mn(II) (as above) and 50 kU L^–1^ superoxide dismutase (SOD; bovine, Sigma) for 1 h followed by Mn oxide quantification.

### Identifying Candidate Mn(II)-Oxidizing Proteins

To identify candidate Mn(II)-oxidizing proteins in the fungal secretomes, we separated the secretomes on gels, excised Mn(II)-oxidizing bands, and sequenced the proteins therein.

#### Protein Electrophoresis and In-Gel Mn(II) Oxidation Assay

Secretome samples from all 3 fungi and 3 time points were separated using standard native PAGE on pre-cast AnyKd^TM^ tris-glycine gels (Bio-Rad) with an average of 10 μg protein loaded per lane, depending on the measured protein concentration of the sample. To retain Mn(II)-oxidizing activity, protein samples were not boiled or denatured. [Note that without protein unfolding, accurate estimates of molecular weight cannot be made, in contrast to traditional SDS-PAGE (Durand et al., 2012)]. Duplicate gels were prepared for all samples: one gel was stained with BioSafe Coomassie G250 (Bio-Rad) for 1 h to visualize all secreted proteins, and the other gel was incubated with 400 μM Mn(II) for 2 h followed by staining with LBB to visualize Mn(II)-oxidizing bands. While brown Mn oxide bands appeared on the gels within minutes after Mn(II) addition, the incubation was allowed to proceed for 2 h to maximize visibility during LBB staining. [Because Mn(II) was not added in excess and it is likely that some samples oxidized all the added Mn(II), quantitative estimates of Mn(II) oxidative capacity cannot necessarily be made based on the intensity of the gel bands]. All gels were rinsed with ultrapure water. LBB-stained Mn(II)-oxidizing bands were excised in a sterile biosafety cabinet, in addition to control bands from lanes run with sample buffer only. Bands were stored at −80°C until analysis via LC/MS/MS.

#### Protein Identification by LC/MS/MS

Peptide sequencing of secretome samples was performed at the Environmental Molecular Sciences Laboratory, part of Pacific Northwest National Laboratory. Four biological replicates of gel band samples (for each species and time point) were analyzed.

##### Preparation of gel band samples

A preliminary comparison of proteins identified in LBB-stained bands and those identified in corresponding Coomassie-stained bands revealed that the presence of LBB did not interfere with LC/MS/MS analysis. Therefore, all gel bands selected for protein analysis were taken from LBB-stained gels to ensure accuracy in identifying Mn(II)-oxidizing bands.

The in-gel digestion procedure was similar to previously described (Shevchenko et al., 2007) and is detailed in the Supplementary Material.

##### LC/MS/MS on gel band samples

The peptide solution was processed on a custom built LC system using two Agilent 1200 nanoflow pumps and one Agilent 1200 cap pump (Agilent Technologies) with various Valco valves (Valco Instruments Co.), and utilizing a PAL autosampler (Leap Technologies) that were fully automated with custom software to allow parallel processing of two columns. Data were acquired using a Velos Orbitrap mass spectrometer (Thermo Scientific) with a custom-made electrospray ionization (ESI) interface. All LC/MS/MS details are provided in the Supplementary Material.

##### Bioinformatics

MS/MS spectra were converted to ASCII text using the DeconMSn tool (Mayampurath et al., 2008) which helps account for incorrect monoisotope assignments. Spectra were then searched with MSGFPlus (Kim and Pevzner, 2014) using a protein FASTA containing sequenced genomes of the 3 fungal species (see details below) and amended with common contaminants (e.g., trypsin and human keratin sequences). All peptide candidate sequences were dynamically modified for oxidized methionine, with a parent mass tolerance of ±20 ppm. Other settings include partially tryptic cleavages, ±1 Da parent corrections (to further account for incorrect monoisotope assignments), MS level data centroiding, and decoy search mode enabled. Results were collated using in-house software and filtered using SQL.

To map identified peptides to associated proteins, peptides from each of the 3 fungi were searched against 3 frame stop-to-stop translations of at least 30 amino acids from their respective genomes, which were still in contig form at the time this work was performed. The number of identified peptides per protein was then summed across 4 biological replicates for each fungus and time point, subtracting out any peptides found in negative control bands. Only proteins with at least 2 peptide observations per band were included in the analysis.

Functional information was obtained by searching the NCBI non-redundant protein database for proteins identified in this study using BLAST. For proteins for which the highest-scoring (highest raw score) match was hypothetical or uncharacterized, the highest-scoring non-hypothetical match was reported. Proteins with no matches (hypothetical or otherwise) having an *E*-value below 10^–10^ were reported as hypothetical. Functional information obtained via BLAST analysis was manually checked against automatically assigned annotations in the JGI-assembled genomes. All proteins were then functionally categorized according to the Carbohydrate-Active Enzymes (CAZy) Database.

The mass spectrometry proteomics data have been deposited to the ProteomeXchange Consortium via the PRIDE partner repository with the dataset identifier PXD021837 and 10.6019/PXD021837.

To validate functional annotation of MCOs identified in the gel bands, a multiple sequence alignment was performed using CLUSTAL Omega (version 1.2.4) against known Ascomycota MCO sequences from GenBank. Sequences included tyrosinases, polyphenol oxidases, bilirubin oxidases, laccasses, and ascorbate oxidases, as well as Ascomycota sequences identified only generally as multicopper oxidases. Conserved Cu-binding domains were identified using NCBI BLAST.

### Validating Candidate Mn(II)-Oxidizing Proteins

We conducted enzyme activity assays on concentrated secretome samples to further validate protein identifications obtained via LC/MS/MS. Specifically, we added enzyme-specific inhibitory chemicals or preferred enzyme substrates to quench enzymatic Mn(II) oxidation. For each assay, 0.5–1.0 μg fungal protein from secretome samples was incubated with 350 μM MnCl_2_ and an inhibitor or preferred substrate in 20 mM HEPES buffer, pH 7.0. Samples were incubated for 1–4 h, depending on initial screening of enzyme activity, in the dark at 21°C, followed by Mn oxide quantification via LBB. All data were normalized to a matrix control containing 0 μM inhibitor or preferred substrate. Assays were conducted with the same 4 biological replicates (for each species and time point) that were used to obtain LC/MS/MS protein identifications. Only selected time points were analyzed due to limited quantities of original secretome samples.

To test for the presence of Mn(II)-oxidizing flavoproteins containing a flavin-adenine dinucleotide (FAD)-binding domain, including FAD oxidoreductases, secretome samples were incubated with 1, 10, or 100 μM of diphenylene iodonium (DPI) in 10% dimethyl sulfoxide (DMSO). DPI inhibits reduction of the FAD domain in flavoproteins (O’Donnell et al., 1993); it can further inhibit superoxide production in FAD-containing NADPH oxidases (Ellis et al., 1988). To test more specifically for the potential Mn(II)-oxidizing activity of glucose-methanol-choline (GMC) oxidoreductases, a superfamily of FAD-containing enzymes (Cavener, 1992), many of which are involved in fungal lignocellulose degradation (Levasseur et al., 2013), samples were incubated with 100, 200, or 400 μM of preferred substrates D-glucose or choline chloride.

To test for the presence of Mn(II)-oxidizing MCOs/LMCOs, samples were incubated with 1, 10, or 100 μM of the copper-chelating compound *o*-phenanthroline in 10% EtOH (Thompson et al., 2006). To test more specifically for the potential Mn(II)-oxidizing activity of tyrosinase, a 2-copper MCO that can oxidize phenols to dopaquinone (Ramsden and Riley, 2014), samples were incubated with 100, 200, or 400 μM of the phenolic amino acid L-tyrosine, a substrate it should prefer over Mn(II). Finally, we tested for the presence of bilirubin oxidase, a 4-copper LMCO similar to fungal laccases, but distinct from laccases in that it can oxidize bilirubin to biliverdin (Pakhadnia et al., 2009). Samples were incubated with 60 μM unconjugated bilirubin in 8.8% DMSO, buffered by 20 mM HEPES pH 8.0, since bilirubin is largely insoluble at pH < 7. Bilirubin remaining in the samples was quantified by measuring spectrophotometric absorbance at 450 nm (Durand et al., 2012) over a 19-h period. Mn(II) oxidation in the presence of bilirubin was not measured, as the yellow color of the bilirubin reagent interfered with the colorimetric (blue) LBB assay (Supplementary Figure 1).

### Statistics

To test for the effects of time or treatment on Mn(II) oxidation in the secretomes, we first evaluated treatment groups for homogeneity of variances using Bartlett’s test or Levene’s test with α = 0.05. For groups with homogeneity of variances, we then conducted a one-way analysis of variance (ANOVA) with α = 0.05, where secretome age, treatment, or concentration of enzyme inhibitor or preferred substrate was the independent variable, and Mn(IV) equivalents as measured by LBB was the dependent variable. The ANOVA was followed by a Tukey–Kramer *post hoc* test. In cases where the homogeneity of variances assumption for ANOVA was not met, we used the non-parametric Kruskal–Wallis *H* test followed by a Dunn–Bonferroni *post hoc* test. In cases where only two groups were compared, we used an independent samples *t*-test with independent and dependent variables as above. All statistical tests were performed in IBM SPSS Statistics (version 25) or Igor Pro (version 8.04).

## Results

### Mn(II) Oxidative Capacity of the Fungal Secretomes

**FIGURE 1:**
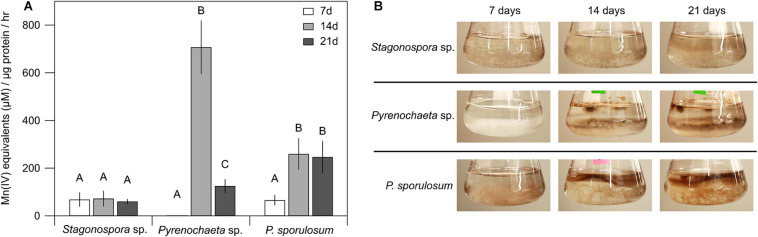
Ascomycete fungi oxidized Mn(II) to Mn(III/IV) oxides over 3 weeks of growth. **(A)** Mn(II) oxidation rate by cell-free fungal secretomes. All experiments were conducted with 500 μM Mn(II) and 200 μg mL^–1^ fungal protein. Samples were incubated with Mn(II) for 1 h prior to Mn oxide quantification. Data were analyzed with a one-way ANOVA followed by a Tukey–Kramer *post hoc* test; within each organism, letters indicate statistically different groups (*N* = 4 for each). Error bars represent ±1 standard deviation. **(B)** Fungal cultures of *Stagonospora* sp., *Pyrenochaeta* sp., and *P. sporulosum* in liquid AY + Mn medium at time of secretome harvest. Dark brown color indicates presence of biogenic Mn oxides. Images are representative of 4 biological replicates.

Patterns of Mn(II) oxidation in the cell-free, >10 kDa secretome were species-specific and varied over time (Figure 1A). Secretome samples from *P. sporulosum* exhibited moderate Mn(II) oxidative capacity early in growth (7 days) that significantly increased (*P* = 4.8 × 10^–3^) in capacity over time. In contrast, while the *Pyrenochaeta* sp. secretome did not oxidize Mn(II) after 7 days (Figure 1B), it exhibited a peak Mn(IV) production rate [708 μM Mn(IV) produced μg protein^–1^ hr^–1^] at 14 days, the highest we observed among the 3 species, and this rate significantly dropped off by 21 days (*P* = 3.9 × 10^–6^). Secretome samples from *Stagonospora* sp. exhibited moderate [61–73 μM Mn(IV) μg^–1^ hr^–1^] Mn(II) oxidative capacity that did not significantly change during the 3-week study (*P* = 0.84). For all 3 fungi, < 10 kDa filtrate remaining after secretome harvest did not exhibit Mn(II) oxidative capacity measurable with LBB (data not shown).

Temporal changes in Mn(II) oxidative capacity were visually evident in the liquid cultures of the fungi prior to biomass removal and secretome harvest (Figure 1B). In *Pyrenochaeta* sp., visible extracellular, dark brown Mn(III/IV) oxides were mainly associated with fungal biomass at 14 and 21 days, with no visible oxides present after 7 days. In *P. sporulosum* cultures, Mn(III/IV) oxides were located predominantly in the growth medium above the biomass and visually increased in abundance from 7 days to 14–21 days. In *Stagonospora* sp. cultures, particulate, spherical Mn(III/IV) oxides that were spatially separated from the biomass were visible at moderate levels across all time points. Thus, visual observations supported the LBB defined quantitative Mn(II) oxidative capacity data. Morphological patterns of Mn(III/IV) oxide formation are in agreement with our previous observations (Santelli et al., 2011) and suggest species-specific Mn(II) oxidation mechanisms.

### Proteinaceous Nature of Mn(II)-Oxidizing Factors

**FIGURE 2:**
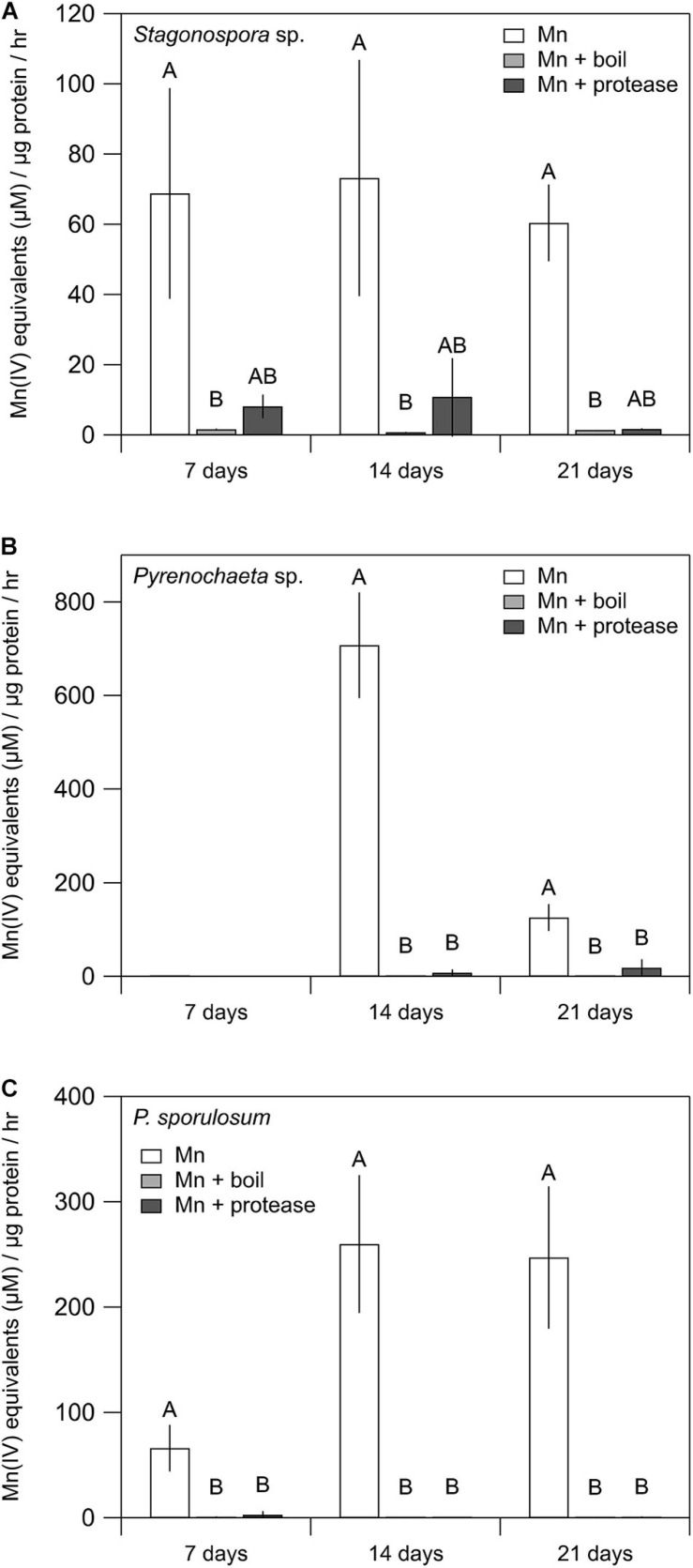
Treating fungal secretomes with heat or protease abolished Mn(II) oxidation. **(A–C)** Mn(II) oxidation rate in the untreated secretome (white bars), after boiling at 100°C for 30 min (light gray bars), and after incubation with 1 mg mL^–1^ proteinase K for 2 h (dark gray bars). For *Stagonospora* sp., data were analyzed with a Kruskal–Wallis *H* test followed by a Dunn–Bonferroni *post hoc* test. For *Pyrenochaeta* sp. and *P. sporulosum*, data were analyzed with a one-way ANOVA followed by a Tukey–Kramer *post hoc* test. Within each organism, letters indicate statistically different groups (*N* = 3 or *N* = 4 for each). Error bars represent ±1 standard deviation.

Mn(II) oxidative capacity in the >10 kDa secretome was nearly or completely abolished by boiling or incubation with proteinase K for all 3 fungi across all time points (Figure 2). Furthermore, the Mn(II) oxidative capacity of the fungi was largely unaffected by the addition of SOD (*P* > 0.05 for each species and time point) (Supplementary Figure 2).

**FIGURE 3:**
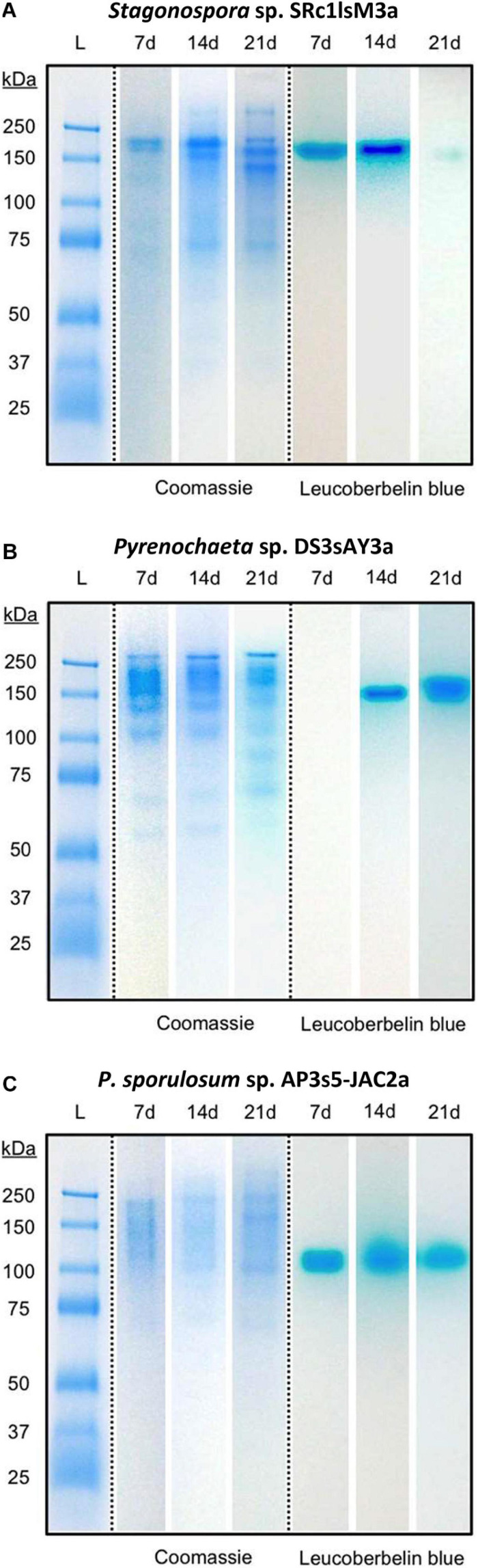
Fungal extracellular enzymes directly oxidized Mn(II). Native PAGE of secretome samples from all 3 time points for **(A)** *Stagonospora* sp., **(B)** *Pyrenochaeta* sp., and **(C)** *P. sporulosum*. Lane L: Pre-stained blue protein molecular mass markers with sizes in kDa to left of images. Center panels: Gel stained with Coomassie G-250 for 1 h. Right panels: Replicate gel of the same secretome samples after incubation with 400 μM Mn(II) for 2 h followed by staining with Leucoberbelin blue, which turns blue in the presence of Mn(III/IV) oxides. Images show 1 biological replicate that is representative of 4 total replicates of each species and time point.

Separating extracellular fungal proteins on native PAGE gels demonstrated that each fungal secretome was rich in protein (as evidenced by Coomassie staining) and exhibited species-specific protein migration patterns (Figure 3, center panels). Moreover, the in-gel Mn(II) oxidation assay revealed that each secretome displayed only one primary Mn(II)-oxidizing band (Figure 3, right panels) and that Mn(II) oxidation did not require any substrates or stimulants other than in-gel proteins and added Mn(II). Within each fungus, Mn(II)-oxidizing bands migrated to a consistent position in the gels over all time points for which Mn(II) oxidative capacity was measured. Note that in the absence of denaturing and reducing agents in these native PAGE gels, protein-protein interactions can be retained during separation, and proteins may separate as multi-protein complexes (D’Amici et al., 2008; Nowakowski et al., 2014). Thus, we do not make any conclusions about protein molecular weight or number of proteins per band solely based on these gels.

### Candidate Mn(II)-Oxidizing Proteins

LC/MS/MS analysis of excised Mn(II)-oxidizing gel bands revealed a diverse suite of redox-active proteins that varied by species and over time (Tables 1–3). Notably, multiple redox-active proteins were identified in each time point for each species. The primary candidate Mn(II)-oxidizing proteins for each species are summarized in Table 4 for comparison. Full lists of identified proteins, including non-redox-active proteins that are unlikely to directly catalyze Mn(II) oxidation, are provided in Supplementary Tables 1–3.

**TABLE 1:** Candidate Mn(II)-oxidizing proteins identified via LC/MS/MS in Mn(II)-oxidizing native PAGE gel bands from *Stagonospora* sp. secretomes.

*Stagonospora* sp. identified protein			Best BLAST hit^1^						Predicted enzyme properties		Identification in Mn(II)-oxidizing native PAGE gel bands					
			
											7 days		14 days		21 days	
			
GenBank accession number	JGI protein ID	JGI protein annotation	GenBank accession number	Protein description	Genome	Raw score	*E*-value	Percent identity	CAZy family^2^	Redox cofactor or active site	Bio reps^3^	Total peptides	Bio reps^3^	Total peptides	Bio reps^3^	Total peptides
OAL06359.1	211028| Stasp1	Tyrosinase	KAF20 23949.1	Di-copper- center- containing protein	*Setomelanomma holmii*	550	0.0	68%	*AA1_3*	Cu	8/8	61	4/4	49	2/2	39
OAL02734.1	119700| Stasp1	Multicopper oxidase	KAF20 31423.1	Cupredoxin^4^	*Setomelanomma holmii*	518	8e-179	81%	*AA1_3*	Cu	8/8	41	3/4	26	–	–
OAL06112.1	323207| Stasp1	Glyoxal oxidase, chitinase, CBM 18	KAF18 42338.1	Carbohydrate binding module family 18^5^	*Cucurbitaria berberidis* CBS 394.84	1146	0.0	83%	*AA5_1, CBM 18*	Cu + radical protein complex	6/8	26	4/4	38	2/2	21
OAK94057.1	330396| Stasp1	Superoxide dismutase (Cu/Zn)	XP_03345 2169.1	Copper/zinc binding superoxide dismutase	*Didymella exigua* CBS 183.55	294	3e-100	95%	–	Cu/Zn	–	–	3/4	9	–	–
OAK99381.1	251441| Stasp1	FAD-binding protein	KAF28 49814.1	FAD binding domain-containing protein^6^	*Plenodomus tracheiphilus* IPT5	883	0.0	75%	–	FAD	1/8	7	–	–	2/2	11
OAL03573.1	291332| Stasp1	FAD-binding protein	KAF20 35237.1	FAD binding domain-containing protein^7^	*Setomelanomma holmii*	1070	0.0	83%	–	FAD	–	–	1/4	3	2/2	8
OAK93932.1	261684| Stasp1	GMC oxidoreductase	OWY4 6284.1	Alcohol oxidase	*Alternaria alternata*	1189	0.0	83%	AA3_3	FAD	–	–	1/4	3	2/2	12
OAK94822.1	260169| Stasp1	Copper amine oxidase	KAF19 15680.1	Copper amine oxidase	*Ampelomyces quisqualis*	65.5	1e-10	77%	–	Cu	–	–	–	–	1/2	5
OAL06123.1	343359| Stasp1	Succinate-semialdehyde dehydrogenase [NAD(P)+]	KAF30 04401.1	Succinate-semialdehyde dehydrogenase NADP+ linked	*Curvularia kusanoi*	977	0.0	90%	–	NAD(P)+/ NAD(P)H	–	–	–	–	1/2	4
OAK96127.1	296929| Stasp1	Superoxide dismutase (Fe/Mn)	KAF20 36800.1	Superoxide dismutase (Fe/Mn)	*Setomelanomma holmii*	450	1e-158	93%	–	Fe/Mn	–	–	–	–	1/2	3

**Only redox-active proteins are shown. All proteins shown were identified with 2 or more peptides. The full list of proteins identified in gel bands (including non-redox-active proteins) is provided in Supplementary Table 1. ^1^Best non-hypothetical protein match in the NCBI non-redundant protein database, excluding matches from the 3 species in this study. ^2^CAZy family obtained from JGI protein annotation (regular text) or annotated manually (italics) using CAZy Database (www.cazy.org). ^3^Denotes number of Mn(II)-oxidizing biological replicates in which protein was detected/total number of Mn(II)-oxidizing biological replicates analyzed. ^4^Also matches closely to phenol oxidase A (Byssothecium circinans, accession KAF1953920.1, raw score = 494, E-value = 7E-170, % identity = 79%) and bilirubin oxidase (Lophiotrema nucula, accession KAF2118746.1, raw score = 442, E-value = 2E-149, % identity = 69%). ^5^Also matches closely to glyoxal oxidase (Ampelomyces quisqualis, accession KAF1913762.1, raw score = 1134, E-value = 0.0, % identity = 81%). ^6^Also matches closely to FAD oxidoreductase (Alternaria gaisen, accession KAB2103633.1, raw score = 882, E-value = 0.0, % identity = 77%). ^7^Also matches closely to FAD/FMN-containing isoamyl alcohol oxidase-like protein MreA (Clathrospora elynae, accession KAF19412121, raw score = 1061, E-value = 0.0, % identity = 83%).**

#### *Stagonospora* sp.

We identified 10 redox-active proteins in *Stagonospora* sp. gel bands (Table 1). Proteins with the most peptide observations (up to 61 per time point) all featured copper-containing active sites, including tyrosinase [CAZy auxiliary activities (AA) family 1; (Levasseur et al., 2013)], a cupredoxin [a diverse superfamily of MCOs; (Savelieff et al., 2008)] (CAZy AA1) that exhibited sequence similarity to phenol oxidase A and bilirubin oxidase in BLAST searches, and glyoxal oxidase (a radical-copper oxidase; CAZy AA5). Multiple sequence alignment with known Ascomycota MCOs and consistency of conserved Cu-binding domains supported the identification of tyrosinase and pointed to bilirubin oxidase as the identity of the cupredoxin (Supplementary Figure 3). Tyrosinase and glyoxal oxidase were abundant across all 3 time points, while the cupredoxin was only identified in 7 and 14 day samples. Proteins with fewer peptide observations generally featured FAD cofactors and were more prominent in later time points. These proteins included unspecified FAD-binding proteins and an alcohol oxidase in the GMC oxidoreductase superfamily (CAZy AA3). Thus, candidate Mn(II)-oxidizing proteins increased in diversity over time.

#### *Pyrenochaeta* sp.

We identified 5 redox-active proteins in *Pyrenochaeta* sp. gel bands (Table 2), all of which had relatively few (maximum of 10) peptide observations per time point. Four of the five proteins featured FAD cofactors. These included two unspecified FAD-binding proteins (one of which exhibited sequence similarity to isoamyl alcohol oxidase; CAZy AA3), a GMC oxidoreductase (CAZy AA3) that may be an alcohol oxidase, and bifunctional solanapyrone synthase. The fifth enzyme was a copper amine oxidase-like protein. The number of identified redox-active proteins decreased over time (4 in 14 day secretomes vs. 2 at 21 days), which corresponded with decreasing Mn(II) oxidative capacity of the *Pyrenochaeta* sp. secretome (Figure 1).

**TABLE 2:** Candidate Mn(II)-oxidizing proteins identified via LC/MS/MS in Mn(II)-oxidizing native PAGE gel bands from *Pyrenochaeta* sp. secretomes.

*Pyrenochaeta* sp. identified protein			Best BLAST hit^1^						Predicted enzyme properties		Identification in Mn(II)-oxidizing native PAGE gel bands					
			
											7 days		14 days		21 days	
			
GenBank accession number	JGI protein ID	JGI protein annotation	GenBank accession number	Protein description	Genome	Raw score	*E*-value	Percent identity	CAZy family^2^	Redox cofactor or active site	Bio reps^3^	Total peptides	Bio reps^3^	Total peptides	Bio reps^3^	Total peptides
OAL54547.1	640056| Pyrsp1	FAD-binding protein	KAF184 6528.1	FAD-binding domain-containing protein	*Cucurbitaria berberidis* CBS 394.84	661	0.0	69%	–	FAD	No Mn(II) oxidation observed in 7 day secretomes		4/4	8	–	–
OAL53386.1	678105| Pyrsp1	GMC oxidoreductase	KAF184 5720.1	GMC oxidoreductase^4^	*Cucurbitaria berberidis* CBS 394.84	1131	0.0	79%	AA3	FAD			–	–	2/3	10
OAL43510.1	524095| Pyrsp1	FAD-binding protein	KAF18 43187.1	Bifunctional solanapyrone synthase	*Cucurbitaria berberidis* CBS 394.84	775	0.0	75%	*AA7*	FAD			2/4	4	1/3	3
OAL51393.1	586913| Pyrsp1	Copper amine oxidase	KAF18 51230.1	Copper amine oxidase-like protein	*Cucurbitaria berberidis* CBS 394.84	1316	0.0	92%	–	Cu			1/4	2	–	–
OAL49332.1	681286| Pyrsp1	FAD-binding protein, berberine-like	KAF18 46047.1	FAD-binding domain-containing protein^5^	*Cucurbitaria berberidis* CBS 394.84	1019	0.0	76%	–	FAD			1/4	2	–	–

**Only redox-active proteins are shown. All proteins shown were identified with 2 or more peptides. The full list of proteins identified in gel bands (including non-redox-active proteins) is provided in Supplementary Table 2. ^1^Best non-hypothetical protein match in the NCBI non-redundant protein database, excluding matches from the 3 species in this study. ^2^CAZy family obtained from JGI protein annotation (regular text) or annotated manually (italics) using CAZy Database (www.cazy.org). ^3^Denotes number of Mn(II)-oxidizing biological replicates in which protein was detected/total number of Mn(II)-oxidizing biological replicates analyzed. ^4^Also matches closely to alcohol oxidase (Decorospora gaudefroyi, accession KAF1838604.1, raw score = 1070, E-value = 0.0, % identity = 75%). ^5^Also matches closely to FAD/FMN-containing isoamyl alcohol oxidase-like protein MreA (Westerdykella ornata, accession XP_033648977.1, raw score = 928, E-value = 0.0, % identity = 68%)*.*

#### P. sporulosum

We identified 7 redox-active proteins in *P. sporulosum* gel bands (Table 3). Two proteins were identified with high peptide observations (up to 52 or 26 per time point) across all 3 time points: (1) a FAD-containing GMC oxidoreductase (CAZy AA3) that exhibited sequence similarity to choline dehydrogenase, and (2) a Cu-containing cupredoxin (CAZy AA1) that exhibited sequence similarity to phenol oxidase A and bilirubin oxidase (the same GenBank entries to which the *Stagonospora* sp. cupredoxin matched). Multiple sequence alignment with known Ascomycota MCOs and Cu-binding domains supported the identification of the cupredoxin as bilirubin oxidase (Supplementary Figure 3). Proteins with fewer peptide observations were only observed in 1 time point, as follows: In 7 day secretomes, we identified a diverse suite of enzymes including 2 peroxidases (potential CAZy AA2), 2-methylcitrate dehydratase (which binds a 4Fe/4S cluster), and a GMC oxidoreductase (CAZy AA3) that may be an alcohol oxidase. In the 21 day secretome, we identified an unspecified FAD-binding protein. Thus, candidate Mn(II)-oxidizing proteins decreased in diversity over time, which contrasts with increasing Mn(II) oxidative capacity of the secretome over time (Figure 1).

**TABLE 3:** Candidate Mn(II)-oxidizing proteins identified via LC/MS/MS in Mn(II)-oxidizing native PAGE gel bands from *P. sporulosum* secretomes.

*P. sporulosum* identified protein			Best BLAST hit^1^						Predicted enzyme properties		Identification in Mn(II)-oxidizing native PAGE gel bands					
			
											7 days		14 days		21 days	
			
GenBank accession number	JGI protein ID	JGI protein annotation	GenBank accession number	Protein description	Genome	Raw score	*E*-value	Percent identity	CAZy family^2^	Redox cofactor or active site	Bio reps^3^	Total peptides	Bio reps^3^	Total peptides	Bio reps^3^	Total peptides
XP_0180 32780.1	1152844| Parsp1	GMC oxidoreductase	KAF24 39767.1	GMC oxidoreductase^4^	*Karstenula rhodostoma* CBS 690.94	1232	0.0	93%	AA3	FAD	4/4	52	4/4	33	3/4	23
XP_01803 0874.1	1129270| Parsp1	Multicopper oxidase	KAF19 71487.1	Cupredoxin^5^	*Bimuria novae-zelandiae* CBS 107.79	201	6e-59	86%	*AA1_3*	Cu	3/4	7	4/4	26	4/4	17
XP_01803 3536.1	961577| Parsp1	Peroxidase, heme-binding	KAF19 65650.1	Heme peroxidase	*Bimuria novae-zelandiae* CBS 107.79	883	0.0	71%	*AA2*	Fe	4/4	21	–	–	–	–
XP_01803 5449.1	827017| Parsp1	2-methylcitrate dehydratase	KAF19 66230.1	2-methylcitrate dehydratase	*Bimuria novae-zelandiae* CBS 107.79	1077	0.0	93%	–	Fe/S	4/4	18	–	–	–	–
XP_01803 5114.1	1165126| Parsp1	GMC oxidoreductase	KAF24 48902.1	GMC oxidoreductase^6^	*Karstenula rhodostoma* CBS 690.94	1046	0.0	87%	AA3_2	FAD	1/4	2	–	–	–	–
XP_01803 5504.1	1165466| Parsp1	Alkyl hydroperoxide reductase, peroxiredoxin	KAF24 46886.1	AhpC/TSA family protein- like protein (thioredoxin-like peroxidase)^7^	*Karstenula rhodostoma* CBS 690.94	335	7e-116	97%	–	Peroxidatic cysteine	1/4	2	–	–	–	–
XP_01803 3970.1	1188260| Parsp1	FAD-binding protein	KAF24 45831.1	FAD-binding domain-containing protein	*Karstenula rhodostoma* CBS 690.94	1009	0.0	94%	–	FAD	–	–	–	–	1/4	2

**Only redox-active proteins are shown. All proteins shown were identified with 2 or more peptides. The full list of proteins identified in gel bands (including non-redox-active proteins) is provided in Supplementary Table 3. ^1^Best non-hypothetical protein match in the NCBI non-redundant protein database, excluding matches from the 3 species in this study. ^2^CAZy family obtained from JGI protein annotation (regular text) or annotated manually (italics) using CAZy Database (www.cazy.org). ^3^Denotes number of Mn(II)-oxidizing biological replicates in which protein was detected/total number of Mn(II)-oxidizing biological replicates analyzed. ^4^Also matches closely to choline dehydrogenase (Decorospora gaudefroyi, accession KAF1830697.1, raw score = 1022, E-value = 0.0, % identity = 77%). ^5^Also matches closely to phenol oxidase A (Byssothecium circinans, accession KAF1953920.1, raw score = 193, E-value = 4E-56, % identity = 84%) and bilirubin oxidase (Lophiotrema nucula, accession KAF2118746.1, raw score = 184, E-value = 8E-53, % identity = 78%). ^6^Also matches closely to alcohol oxidase (Bimuria novae-zelandiae CBS 107.79, accession KAF1969415.1, raw score = 943, E-value = 0.0, % identity = 77%). ^7^Also matches closely to peroxiredoxin-2D (Periconia macrospinosa, accession PVI06010.1, raw score = 313, E-value = 4E-107, % identity = 90%).**

**TABLE 4:** Summary of primary candidate Mn(II)-oxidizing enzymes in the secretomes of 3 filamentous Ascomycete fungi.

			
Species	Secretome age		
	
	7 days	14 days	21 days
			
*Stagonospora* sp.	Tyrosinase (Cu)		
	Bilirubin oxidase (Cu)		
	Glyoxal oxidase (Cu)		
	GMC oxidoreductase (FAD) and other FAD-binding proteins		
*Pyrenochaeta* sp.		GMC oxidoreductase (FAD) and other FAD-binding proteins	
		Cu amine oxidase (Cu) and/or other metalloenzymes?	
*P. sporulosum*	GMC oxidoreductase (FAD)		
	Bilirubin oxidase (Cu)		

**For each enzyme, the redox cofactor or active site is noted in parentheses*.*

### Validation of Candidate Mn(II)-Oxidizing Proteins

**FIGURE 4:**
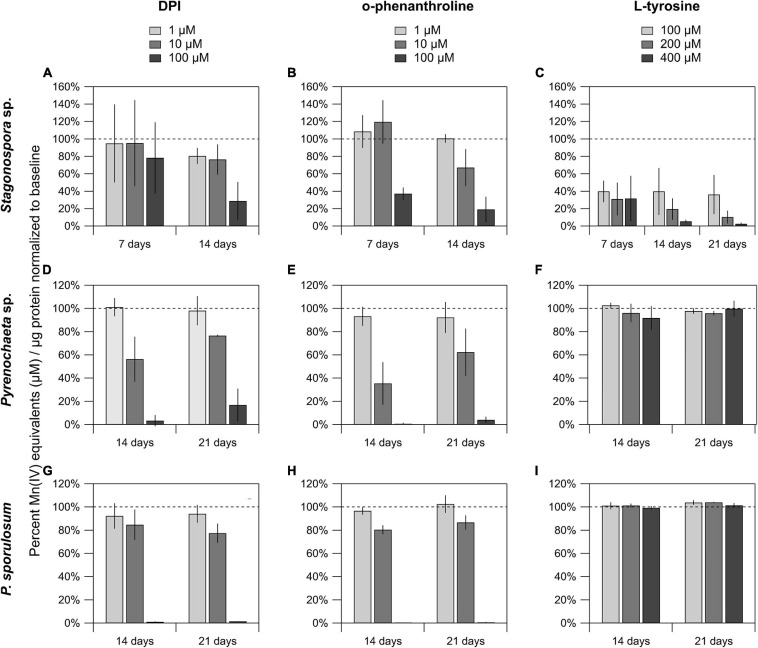
Mn(II) oxidation was inhibited by DPI and *o*-phenanthroline in the secretomes of all 3 fungi, and by L-tyrosine in *Stagonospora* sp. Mn(II) oxidation in cell-free secretomes of **(A–C)** *Stagonospora* sp., **(D–F)** *Pyrenochaeta* sp., and **(G–I)** *P. sporulosum* in the presence of 1, 10, or 100 μM DPI in 10% DMSO (left) or *o*-phenanthroline in 10% EtOH (center), or 100, 200, or 400 μM L-tyrosine (right) in selected time points. All samples were supplemented with 350 μM Mn(II) and incubated for 1 h before Mn oxide quantification, except for L-tyrosine assays in *Stagonospora* sp. which were incubated for 4 h due to low enzyme activity after an initial 1 h trial. All data are normalized to a matrix control with 0 μM reagent. Error bars represent ±1 standard deviation (*N* = 3 for *Stagonospora* sp. and *Pyrenochaeta* sp.; *N* = 4 for *P. sporulosum*).

The FAD inhibitor DPI substantially reduced Mn(II) oxidation across all 3 species for all time points analyzed, with the highest concentration (100 μM) nearly or completely abolishing Mn(II) oxidation in the *Pyrenochaeta* sp. and *P. sporulosum* secretomes (Figures 4D,G). In *Stagonospora* sp. samples, inhibitory effects of DPI were observed at 14 days but not 7 days (Figure 4A). GMC oxidoreductase substrates glucose and choline, in concentrations up to 400 μM, had no measurable effect on Mn(II) oxidation in any of the 3 species (Supplementary Figure 4).

Similar to DPI, widespread decreases in Mn(II) oxidation were observed with Cu-chelating *o*-phenanthroline (Figures 4B,E,H), which had a greater impact in *Stagonospora* sp. than DPI, including in the 7 day samples. Tyrosinase substrate L-tyrosine substantially quenched Mn(II) oxidation in all 3 *Stagonospora* sp. time points (Figure 4C) but had no effect in the other 2 fungi (Figures 4F,I). Small quantities of unconjugated bilirubin were oxidized in all 3 species (Supplementary Figure 5), but oxidation was only statistically significant in 21 day *P. sporulosum* secretomes (*P* = 0.028), which demonstrated consistent oxidation of bilirubin over a 19-h period (Supplementary Figure 5C).

## Discussion

This study demonstrated species-specific, age-dependent, and enzyme-directed Mn(II) oxidative capacity in the secretomes of 3 filamentous Ascomycete fungi. We also identified a diverse suite of candidate Mn(II)-oxidizing enzymes in each organism.

### Hypothesis 1: Mn(II) Oxidation Is Directly Catalyzed by Extracellular Proteins in the Fungal Secretomes

In support of our hypothesis, here we demonstrated extracellular Mn(II) oxidative capacity in the cell-free secretomes of three phylogenetically diverse, filamentous Ascomycete fungi (Figure 1). This capacity was abolished by boiling and incubation with a protease (Figure 2). While boiling could reduce the activity of reactive metabolites in the secretome, the action of proteases is specific to proteins. Thus, Mn(II) oxidative capacity in the secretomes was mediated by extracellular proteins. Our findings support previous observations of Mn(II) oxidative capacity in the extracellular secretome in diverse Mn(II)-oxidizing microorganisms, including Ascomycete fungi (Miyata et al., 2004), Basidiomycete fungi (Glenn and Gold, 1985; Glenn et al., 1986), bacteria (Learman et al., 2011a), and green algae and cyanobacteria (Chaput et al., 2019).

We further demonstrated that extracellular Mn(II) oxidation was not significantly affected by SOD (Supplementary Figure 2), indicating that the catalytic action of extracellular enzymes, rather than superoxide potentially produced by these enzymes, drove Mn(II) oxidation. Thus, cell-free oxidative capacity in the secretome represents a distinct Mn(II) oxidation mechanism from the hyphal-associated, superoxide-mediated mechanism we have previously observed for two of these organisms (*Stagonospora* sp. and *Pyrenochaeta* sp.) (Tang et al., 2013) and a third fungus, *Stilbella aciculosa* (Hansel et al., 2012), during growth on agar-solidified medium. While transmembrane NADPH oxidases (and therefore vegetative cells) are required for superoxide production and Mn(II) oxidation at the cell surface, here we showed Mn(II) oxidation in the absence of cells and these membrane-associated enzymes. Moreover, we demonstrated that extracellular enzymes could oxidize Mn(II) in rinsed native PAGE gels (Figure 3) in the absence of other reactive metabolites that were present in the full secretomes. This indicates that the enzymes can directly utilize Mn(II) as a substrate.

Enzymatic Mn(II) oxidation in the cell-free secretomes exhibited species-specific temporal patterns over the 3-week study (Figure 1A), similar to time-dependent oxidation dynamics observed in Mn(II)-oxidizing bacteria (Jung and Schweissfurth, 1979; Francis et al., 2001) and phototrophs (Chaput et al., 2019). These patterns suggest that secretion of Mn(II)-oxidizing proteins may be linked to species-specific growth dynamics, potential physiological roles of Mn(II) oxidation, or environmental conditions such as substrate availability. The observed delay in the onset of Mn(II) oxidative capacity in *Pyrenochaeta* sp. and the increase over time in *P. sporulosum* (Figure 1A) could be explained by age-dependent enzyme secretion dynamics that may be associated with reduced substrate availability, particularly after 2–3 weeks in small batch cultures. Indeed, preliminary analysis of the *Pyrenochaeta* sp. secretome via nuclear magnetic resonance (NMR) spectroscopy revealed that acetate, the main C source in AY+Mn medium, had been depleted by 14 days (Supplementary Figure 6), corresponding with the onset of Mn(II) oxidative capacity. This raises the possibility of a physiological role for Mn(II) oxidation in the breakdown of existing fungal biomass to recycle C and nutrients, similar to Basidiomycete fungi that harness Mn(II) oxidation for C acquisition in lignocellulose (Dashtban et al., 2010). Previous research has found that Mn(II) oxidation may be induced by starvation or the onset of stationary phase in Mn(II)-oxidizing bacteria *P. putida* strains MnB1 and GB-1 (Jung and Schweissfurth, 1979; Okazaki et al., 1997) and *Erythrobacter* strain SD-21 (Francis et al., 2001).

Temporal patterns in the cell-free secretomes may also be driven by the interaction of enzymatic and abiotic mechanisms, such as initial enzymatic Mn(II) oxidation followed by abiotic mineral surface-catalyzed oxide formation. This could explain the steady increase in visual Mn oxide formation in *Pyrenochaeta* sp. cultures (Figure 1B) despite a marked decrease in enzymatic Mn(II) oxidative capacity at 21 days (Figure 1A), presumably driven by reduced enzyme secretion. In liquid cultures containing live biomass, age-dependent superoxide production by hyphal-associated NADPH oxidases could also contribute to oxide formation patterns. Taken together, Mn(II) oxidative capacity may be a function of the changing composition of the secretome over time, including enzymes, minerals, and metabolites. Future research is needed to determine any involvement of minerals and metabolites in the enzymatic processes we describe here.

### Hypothesis 2: Extracellular Mn(II)-Oxidizing Proteins Vary by Species and Over Time as a Function of Secretome Composition

In support of our hypothesis, we identified a diverse suite of redox-active proteins in Mn(II)-oxidizing gel bands that varied by species and over time (Tables 1–4). To our surprise, we identified multiple redox-active proteins in each time point for each species, which suggests that multiple Mn(II)-oxidation mechanisms are operative in each species, and that these mechanisms vary with secretome age. Our findings are similar to a study of the Mn(II)-oxidizing bacterium *Pseudomonas putida* GB-1, in which the authors posited that the bacterium may have multiple Mn(II)-oxidizing enzymes that dominate under different growth conditions (Geszvain and Tebo, 2010). Indeed, we found strong evidence consistent with both FAD-binding and copper-containing Mn(II)-oxidizing enzymes in all 3 species (Figure 4), demonstrating mechanistic redundancy in these fungi. We propose that the identified proteins serve as candidate Mn(II)-oxidizing enzymes that can be used as targets for future enzyme isolation, purification, and biochemical characterization studies.

#### Tyrosinase

We identified tyrosinase as a strong candidate Mn(II)-oxidizing enzyme in *Stagonospora* sp., but not the other 2 species (Table 4). To support this, we demonstrated that secretome-based Mn(II) oxidative capacity was strongly inhibited by 100 μM of the Cu-chelator *o*-phenanthroline and by 100–400 μM L-tyrosine (Figure 4) across all 3 time points. Both types of inhibition exhibited concentration dependence. Thus, our data point to a Mn(II)-oxidizing metalloenzyme that is inhibited by L-tyrosine. In the other 2 species, we neither identified tyrosinase in the LC/MS/MS data, nor did we observe L-tyrosine-based inhibition of Mn(II) oxidation.

While our identification of tyrosinase is supported by alignment of conserved Cu-binding domains with other Ascomycete tyrosinases and polyphenol oxidases (Supplementary Figure 3), the inhibition assays are less definitive. Although inhibition by *o*-phenanthroline is consistent with a Cu-containing enzyme, it does not definitively indicate the presence of Cu because *o*-phenanthroline can chelate other metals, including Mn (Brandt et al., 1954). It can also inhibit the activity of other Mn(II)-oxidizing metalloenzymes that do not contain Cu, such as the Mn(II)-oxidizing heme peroxidase in *Erythrobacter* SD-21 (Francis et al., 2001; Johnson and Tebo, 2008). Likewise, inhibition of Mn(II) oxidative capacity by L-tyrosine does not necessarily implicate tyrosinase in Mn(II) oxidation. Like tyrosinase, fungal laccases (4-Cu MCOs) can also oxidize phenols including L-tyrosine (Mattinen et al., 2005) and could be responsible for the L-tyrosine-mediated inhibition of Mn oxide formation we observed here.

Tyrosinase has a bicopper active site and can generate *ortho*-quinones from phenols (such as L-tyrosine) and catechols (Ramsden and Riley, 2014). Although direct Mn(II) oxidation by tyrosinase has not been established, mushroom tyrosinase can oxidize Fe(II) to Fe(III) (Boyer et al., 1986). In some tyrosinases, oxidation of L-tyrosine is inhibited by Mn(II) (Palumbo et al., 1985). In our study, we observed the opposite effect, in which oxidation of Mn(II) was inhibited by L-tyrosine. Thus, competitive inhibition could be occurring, in which tyrosinase produced by *Stagosnopora* sp. could utilize both Mn(II) and L-tyrosine as substrates, but both may not be able to bind simultaneously. Further research is warranted to determine whether tyrosinase can indeed directly oxidize Mn(II), and if so, how.

#### Bilirubin Oxidase

We identified a MCO with a cupredoxin fold as a strong candidate Mn(II)-oxidizing enzyme in both *Stagonospora* sp. (in 7 and 14 day secretome samples) and in *P. sporulosum* (all 3 time points) (Table 4). In both species, the cupredoxins exhibit 70–80% sequence similarity to phenol oxidase A (polyphenol oxidase or tyrosinase) and bilirubin oxidase based on NCBI BLAST searches (Tables 1, 3), and the cupredoxins from both species map to the same GenBank entries. Multiple sequence alignment with other Ascomycota MCOs suggests the enzymes from both species are bilirubin oxidase (Supplementary Figure 3). Consistent with this Cu-containing metalloenzyme, we identified strong, concentration-dependent inhibition of Mn(II) oxidation by *o*-phenanthroline in both species (Figure 4).

In *P. sporulosum*, we only identified 1 metal-containing, redox-active protein (accession XP_018030874.1, the potential bilirubin oxidase) in the gel bands with high peptide observations in all time points in which *o*-phenanthroline inhibition was observed. Furthermore, in *P. sporulosum*, the lack of inhibition by L-tyrosine (Figure 4) suggests that tyrosinase or other polyphenol oxidases were not driving Mn(II) oxidation in this species, in contrast to *Stagonospora* sp. Therefore, bilirubin oxidase was likely the major contributor to a metalloenzyme-based Mn(II) oxidation mechanism in *P. sporulosum* (Table 4). In *Stagonospora* sp., Mn(II) oxidative capacity was lowest at 21 days (Figure 1A, although specific activities were not statistically different; Figure 3A), and bilirubin oxidase was not identified in 21 day gel bands (Table 1). This could suggest this enzyme conferred a substantial portion of Mn(II) oxidative capacity in this species.

Oxidation of unconjugated bilirubin by the secretomes of both species further supported the identification of bilirubin oxidase. In *Stagonospora* sp., bilirubin oxidation by the secretome was measurable, particularly at 14 days, but was not statistically different from controls (Supplementary Figure 5). In *P. sporulosum*, we observed statistically significant oxidation of unconjugated bilirubin in the 21 day secretome (Supplementary Figure 5). Oxidation of bilirubin to biliverdin is exclusive to bilirubin oxidase as opposed to other MCOs (Pakhadnia et al., 2009).

It is intriguing that potential bilirubin oxidases were identified across 2 Ascomycete species in this study. This finding adds to previous identifications of Mn(II)-oxidizing bilirubin oxidases in the fungus *Acremonium* sp. (Tojo et al., 2020) and the bacterium *L. discophora* (Corstjens et al., 1997), suggesting that Mn(II) oxidation by microbial bilirubin oxidases may be widespread. This enzyme is a 4-Cu LMCO that is classified in the CAZy AA1 (MCO) family along with traditional laccases (Levasseur et al., 2013), a group of enzymes known to oxidize Mn(II) to Mn(III) in white-rot Basidiomycetes (Höfer and Schlosser, 1999). An important limitation in our study is that we did not definitively link bilirubin oxidase to Mn(II) oxidation in enzyme validation assays. Further work is warranted to determine whether bilirubin oxidase in these 2 species can directly oxidize Mn(II).

Taken together, our findings of tyrosinase and bilirubin oxidase as candidate Mn(II)-oxidizing enzymes support a long-established role of MCOs in microbial Mn(II) oxidation, including both fungi (Höfer and Schlosser, 1999) and bacteria (Tebo et al., 2004).

#### Glyoxal Oxidase

We identified glyoxal oxidase, a radical-copper oxidase, with high peptide counts across all 3 time points in *Stagonospora* sp. only (Tables 1, 4). Our observations that *Stagonospora* sp. Mn(II) oxidative capacity was strongly inhibited by a Cu-chelator support potential Mn(II) oxidation by this enzyme, but a direct link to Mn(II) oxidation has not yet been identified. Glyoxal oxidase is classified in the CAZy AA5 family with broad substrate specificity (Whittaker et al., 1996; Whittaker and Whittaker, 1998). Because it is capable of generating H_2_O_2_ from O_2_, it is thought to work cooperatively with type II peroxidases, including Mn peroxidases that oxidize Mn(II) to Mn(III), in lignocellulose degradation by Basidiomycete fungi (Kersten and Cullen, 2014). Purified glyoxal oxidase from the Basidiomycete *Phanerochaete chrysosporium* has been shown to reduce Mn(III) to Mn(II) (Whittaker et al., 1996), but it is unknown whether the activated enzyme can then oxidize Mn(II) back to Mn(III) coupled to the reduction of another electron acceptor, such as O_2_.

#### GMC Oxidoreductase and Other FAD-Binding Proteins

Our data point to Mn(II) oxidation by FAD-containing proteins in all 3 Ascomycete fungi across all time points in which any Mn(II) oxidation was detected (Table 4). We observed strong, concentration-dependent inhibition of Mn(II) oxidative capacity by DPI across all 3 species, with the exception of the *Stagonospora* sp. 7 day secretome (Figure 4). This was consistent with higher peptide counts of FAD-binding proteins in the 14–21 day secretomes in *Stagonospora* sp. than at 7 days (Table 1), suggesting that a FAD-based Mn(II) oxidation mechanism may have been more active as the secretome aged.

Based on BLAST searches of the NCBI database, the identities of the FAD-binding proteins remain poorly resolved across all 3 species. The FAD-binding proteins from the fungi in this study exhibit higher sequence similarity with each other than with known Ascomycete FAD-containing proteins, making identification challenging. GMC oxidoreductase substrates glucose and choline did not inhibit Mn(II) oxidative capacity in any of the 3 species (Supplementary Figure 4), suggesting that the proteins are not glucose oxidase or choline dehydrogenase, although we cannot rule out this possibility. Further characterization of these proteins is warranted to identify their function and their role in Mn(II) oxidation.

In *Stagonospora* sp., 3 primary enzymes were identified: 2 unspecified FAD-binding proteins and an alcohol oxidase in the GMC oxidoreductase superfamily (Table 1). GMC oxidoreductases comprise a large and diverse superfamily of redox-active, H_2_O_2_-generating enzymes, many of which are represented in the CAZy AA3 family (Levasseur et al., 2013). They contain a highly-conserved FAD-binding domain (Zamocky et al., 2004), generally stripping two electrons from a broad range of substrates and reducing O_2_. Their interactions with transition metals have not been well characterized.

In the *P. sporulosum* secretome, GMC oxidoreductase was identified with high peptide observations in all 3 time points (Table 3), making this a strong candidate Mn(II)-oxidizing enzyme in this species. This protein (accession XP_018032780.1) exhibited 77% sequence identity to choline dehydrogenase (Table 3). Choline dehydrogenase is poorly characterized across all domains of life, particularly in fungi, due to difficulty in enzyme purification (Salvi and Gadda, 2013). Its redox cofactor may consist of FAD, pyrroloquinoline quinone (PQQ) (Ameyama et al., 1985; Gadda and McAllister-Wilkins, 2003), an iron/sulfur cluster, or a combination of these (Huang and Lin, 2003).

In *Pyrenochaeta* sp., LC/MS/MS data suggest that FAD-binding proteins were the primary contributors to Mn(II) oxidation in the 14 and 21 day secretomes (Table 2). The only non-FAD-containing, redox-active protein identified in the gel bands was a copper amine oxidase, but only in the 14 day secretome. Although all proteins had relatively low peptide observations in this species, the enzymes in Table 2 were the only redox-active proteins we identified in the gel bands, and thus we conclude they could be involved in Mn(II) oxidation. Two identified FAD-binding proteins (accessions OAL53386.1 and OAL49332.1) exhibited 75 and 68% sequence similarity, respectively, to alcohol oxidases based on BLAST searches (Table 2). Bifunctional solanapyrone synthase, also identified in this species, is an FAD oxidoreductase that has been classified in the CAZy AA7 family of glucooligosaccharide oxidases (Mahajan et al., 2016) and contributes to biosynthesis of the toxin solanapyrone in phytopathogenic fungi (Oikawa et al., 1998). Its interactions with transition metals are not well understood.

FAD-based Mn(II) oxidation by hyphal-associated NADPH oxidases has been previously demonstrated in 3 Ascomycete fungi (including *Stagonospora* sp. and *Pyrenochaeta* sp.) growing on agar-solidified medium (Hansel et al., 2012; Tang et al., 2013). In these cases, extracellular superoxide produced by the NADPH oxidases was the oxidant of Mn(II). Here we demonstrated that extracellular Mn(II) oxidation in the secretome was not impacted by SOD in any of the 3 species (Supplementary Figure 2). Therefore, while the secretomes might have contained FAD oxidoreductases that generated superoxide and were inhibited by DPI, superoxide was not driving Mn(II) oxidation in the secretomes. Our data suggest the presence of a separate FAD-based Mn(II) oxidation mechanism, potentially involving direct enzymatic oxidation of Mn(II). Thus, we have observed an FAD-based microbial Mn(II) oxidation mechanism that does not involve superoxide in all 3 species.

It is possible that FAD-containing enzymes could generate OH radical (^•^OH), which could catalyze the one-electron oxidation of Mn(II) to Mn(III) (van Genuchten and Peña, 2017). This reaction is thermodynamically favorable above pH 1 (Luther, 2010) and could proceed as follows:

Mn(II)+OH•+2HO2→MnOOH+OH+-3H+

Many GMC oxidoreductases are considered to be key producers of the extracellular H_2_O_2_ needed by lignolytic peroxidases in white-rot Basidiomycetes. As such, these enzymes can also contribute to ^•^OH production via Fenton chemistry in the presence of ferrous iron [reviewed in Dashtban et al. (2010)]. While we have not investigated this in our study, these processes could explain a FAD-based Mn(II)-oxidation mechanism in whole secretome samples.

#### Other Enzymes

Numerous other redox-active proteins were identified in the fungal secretomes with low peptide observations that could have supplemented Mn(II) oxidation by the primary candidate enzymes discussed above.

Copper amine oxidases, identified in the *Stagonospora* sp. 21 day secretome and the *Pyrenochaeta* sp. 14 day secretome, are widely distributed among filamentous fungi (Frebort et al., 1997) and have been shown to generate H_2_O_2_ (Tavladoraki et al., 2016). Thus, they could contribute to a potential Fenton-based Mn(II) oxidation mechanism, as discussed above. They could also be inhibited by *o*-phenanthroline, which quenched Mn(II) oxidation in both species.

Interestingly, our data suggest that metalloenzymes such as copper amine oxidase might have been more active in Mn(II) oxidation in the *Pyrenochaeta* sp. secretome than the LC/MS/MS data indicated, perhaps as active as an FAD-based mechanism. While we only identified 1 Cu-containing enzyme in the 14 day gel bands (Table 3), we observed strong inhibition of Mn(II) oxidative capacity by *o*-phenanthroline in both the 14 and 21 day secretomes (Figure 4). This indicates that other Mn(II)-oxidizing metalloenzymes were present in the secretome, particularly at 21 days, that were not captured in our LC/MS/MS data. Lack of inhibition by L-tyrosine (Figure 4) and lack of significant bilirubin oxidation (Supplementary Figure 5) in the 21 day secretome suggests that these metalloenzymes were not tyrosinase or bilirubin oxidase. This further underscores the need for additional studies to definitively identify which proteins are active in Mn(II) oxidation in this species.

Heme peroxidase and 2-methylcitrate dehydratase were both identified in *P. sporulosum* gel bands from the 7 day secretome. Heme peroxidases have been implicated in bacterial Mn(II) oxidation via direct enzymatic catalysis (Dick et al., 2008; Anderson et al., 2009) and through the production of extracellular superoxide (Andeer et al., 2015). 2-methylcitrate dehydratase is involved in propanoate metabolism and binds a 2Fe-2S cluster (Mason and Cammack, 1992). Finally, an AhpC/TSA family protein was identified in the 7 day gel bands; this enzyme is a thioredoxin-like peroxidase with a cysteine sulfenic acid (-SOH) active site. Enzymes of this type have been implicated in *Saccharomyces cerevisiae* in regulating Mn(II) homeostasis (Farcasanu et al., 1999) and as an antioxidant enzyme that protects Mn-SOD from oxidative degradation (Pedrajas et al., 2010).

Two superoxide dismutases were identified in the *Stagonospora* sp. gel bands, each in only 1 time point. It is unlikely that SODs were contributing to Mn(II) oxidation in the secretomes because the addition of exogenous SOD did not significantly affect Mn(II) oxidative capacity in assays in which more than 50% of added Mn(II) remained available for oxidation (Supplementary Figure 2). Succinate-semialdehyde dehydrogenase, also identified in *Stagonospora* sp., is involved in amino acid degradation and reduces NAD(P)+ to NAD(P)H (Takaya, 2009). Any potential role in Mn(II) oxidation remains unclear.

### Data Limitations

While our study demonstrated direct, enzymatic Mn(II) oxidation in the cell-free secretomes of 3 Ascomycete fungi and identified a diverse suite of candidate Mn(II)-oxidizing proteins, it did not directly implicate any individual enzymes in Mn(II) oxidation. Mn(II) oxidative capacity was measured in native PAGE gels in which multiple enzymes were present in Mn(II)-oxidizing bands, and in whole secretome samples that contained the full complement of secreted fungal enzymes and metabolites. It is very probable that some Mn(II)-oxidizing enzymes present in the secretomes were not captured in our LC/MS/MS data, particularly for any proteins that were low in abundance. Any such missed proteins could still have high Mn(II)-oxidizing activity. As discussed earlier, our LC/MS/MS data are clearly incomplete for the 21 day *Pyrenochaeta* sp. secretome. Similarly, identification of redox-active proteins in the gel bands does not necessarily implicate them in Mn(II) oxidation. While our enzyme validation assays supported some protein identifications and their potential role in Mn(II) oxidation, isolation and purification, if possible, of the candidate Mn(II)-oxidizing enzymes will ultimately be required to confirm that these enzymes can act directly on Mn(II).

Direct enzymatic Mn(II) oxidation in the laboratory gel environment may not be representative of processes occurring in the complex environment of the secretome, particularly when fungal biomass was also present. Here, other mechanisms could have been operative in addition to, or instead of, a direct enzymatic mechanism, including a hyphal-associated, superoxide-mediated mechanism. In the whole secretome, organics and other reductants were likely available for ROS production, and therefore, the enzymes identified in this study may indirectly mediate Mn(II) oxidation via production of reactive intermediates. For example, laccases secreted by the Basidiomycete *Stropharia rugosoannulata* can generate organic radicals, such as CO_2_^•–^, through oxidation of reduced organic carbon compounds in the secretome (Schlosser and Höfer, 2002). This organic radical can then react with O_2_ to produce the superoxide radical, which can directly oxidize Mn(II). Similarly, enzymes that can generate H_2_O_2_, including tyrosinase (Ramsden and Riley, 2014), glyoxal oxidase (Kersten and Kirk, 1987), and GMC oxidoreductases (Zamocky et al., 2004), may indirectly oxidize Mn(II) via OH radical production formed via the Fenton reaction between iron and H_2_O_2_. Our study did not investigate these ROS-based mechanisms. Finally, our study did not tease apart complex interactions among enzymes that might have been occurring in the secretome, which could have generated Mn(II) oxidative capacity through other mechanisms.

## Conclusion

Here, we have demonstrated species-specific, age-dependent, and enzyme-directed Mn(II) oxidative capacity in the secretomes of three filamentous Ascomycete fungi. We identified enzymatic Mn(II) oxidation mechanisms in the liquid secretome that are distinct from the hyphal-associated, superoxide-mediated mechanism previously observed on solid substrate (Hansel et al., 2012; Tang et al., 2013). The proteomic composition of Mn(II)-oxidizing gel bands from each of the fungi, in combination with enzyme activity assays, revealed the presence of both Cu-based and FAD-based Mn(II) oxidation mechanisms in all 3 species, demonstrating mechanistic redundancy. Furthermore, the FAD-based mechanisms did not involve superoxide, in contrast to previously observed microbial Mn(II) oxidation by NADPH oxidases (Hansel et al., 2012; Tang et al., 2013). Specifically, we identified candidate Mn(II)-oxidizing enzymes as tyrosinase, bilirubin oxidase, glyoxal oxidase, and GMC oxidoreductase.

The diversity of the candidate Mn(II)-oxidizing enzymes identified in the 3 Ascomycetes suggests that the ability of fungal secretomes to oxidize Mn(II) may be widespread and extend to phylogenetically diverse fungi that secrete these oxidative enzymes. Isolation, purification, and biogeochemical investigation of the enzymes identified herein will further elucidate fungal Mn(II) oxidation mechanisms, aid in identifying a physiological role for Mn(II) oxidation in Ascomycetes, and expand our knowledge of the drivers of Mn redox cycling in the environment and its role in the global carbon cycle.

## Data Availability Statement

The mass spectrometry proteomics data have been deposited to the ProteomeXchange Consortium via the PRIDE partner repository with the dataset identifier PXD021837 and 10.6019/PXD021837.

## Author Contributions

CZ, SW, LP-T, and CH: study conception and design. CZ, EZ, DC, and CS: data collection. CZ, SP, and CH: data analysis. CZ, CS, and CH: data interpretation. CZ, SP, EZ, and CH: manuscript drafting. All authors: critical revisions to the manuscript.

## Conflict of Interest

The authors declare that the research was conducted in the absence of any commercial or financial relationships that could be construed as a potential conflict of interest.
