# Supplementary material for: Mechanisms of Manganese(II) Oxidation by Filamentous Ascomycete Fungi Vary With Species and Time as a Function of Secretome Composition
Source: Front Microbiol. 2021 Feb 10;12:610497. doi: 10.3389/fmicb.2021.610497 (PMC7902709; doi:10.3389/fmicb.2021.610497)
Supplement: Supplementary file 1 [file Data_Sheet_1.docx]

**SUPPLEMENTARY MATERIAL**

**Supplementary Methods**

# Culture conditions and secretome harvesting

To prepare cell-free secretomes, fungi were grown in 100 mL liquid cultures in AY + Mn medium using blender-homogenized inocula. Inocula were prepared by aseptically removing the entire contents of a 90 mm petri dish (including fungal mycelia and associated agar) that had incubated at room temperature (21°C) until the mycelia had reached the edge of the agar. The contents were then placed in an autoclaved kitchen blender (Oster model BVLB07) with 100 mL of AY + Mn medium and homogenized on high speed for 2 minutes. On the same day that the homogenized inocula were prepared, 100 μL of the inoculum was used to inoculate 100 mL liquid cultures.

Cultures were incubated at 21°C without agitation for 7, 14, or 21 days. For each fungus at each of the 3 time points, 5 individual 100 mL cultures were combined into 500 mL samples to maximize protein recovery. These 500 mL samples were prepared in quadruplicate to account for the inherent unpredictability of fungal cultures. Upon harvesting, bulk biomass was removed with a sterile wooden stick and discarded, and the spent medium was filtered through a 0.45 μm polyethersulfone membrane (VWR) to remove remaining cells and Mn oxides. Samples were then concentrated using a centrifugal filter with a 10 kDa, low protein adhesion membrane (EMD Millipore). Centrifugation proceeded at 2200 × g on a Sorvall RT 6000B centrifuge with H1000B swing-bucket rotor until all liquid had passed through the membrane. The resulting secretome samples were rinsed with 20 mM HEPES, pH 7 and extracted from the centrifugal filter by centrifuging upside down, according to the maufacturer’s instructions. These concentrated ~250 µL secretome samples were then stored at -80°C until analysis.

# Preparation of gel band samples

The in-gel digestion procedure was similar to previously described (Shevchenko et al., 2007). Briefly, the excised gel bands were cut into small pieces and were destained with 100 mM ammonium bicarbonate/acetonitrile (1:1, vol/vol) for 30 minutes before drying in acetonitrile. The gel slices were then saturated with a 1.5 µM solution of trypsin in 50 mM ammonium bicarbonate, pH 8.0 at 4°C for 1 hour, then incubated for 1 hour at 58°C with 1000 rpm shaking. The reaction was quenched with 50% formic acid in water, and the peptides were extracted with a 1:2 (vol/vol) 5% formic acid/acetonitrile solution (incubated at 37°C for 15 minutes followed by centrifugation). The extraction was repeated, and the combined supernatants were concentrated in a vacuum concentrator to 100 µL. After ultracentrifugation at 100k rpm on a Beckman TLA 120.1 120K RPM rotor, the resulting supernatant was further concentrated to 30 uL and transferred to an autosampler compatible vial for LC/MS/MS analysis.

# LC/MS/MS on gel band samples

The peptide solution was processed on a custom built LC system using two Agilent 1200 nanoflow pumps and one Agilent 1200 cap pump (Agilent Technologies) with various Valco valves (Valco Instruments Co.), and utilizing a PAL autosampler (Leap Technologies) that were fully automated with custom software to allow parallel processing of two columns. The reversed-phase columns (40 cm x 360 µm o.d. x 75 µm i.d fused silica (Polymicro Technologies Inc.)) were packed in-house with 3 µm Jupiter C18 (Phenomenex). A 1 cm sol-gel frit was used for media retention and a 4-cm length, 5 µm Jupiter C18 trapping column with a frit on both ends were also utilized (Maiolica et al., 2005). The mobile phases were 0.1% formic acid in water (A) and 0.1% formic acid in acetonitrile (B) and were processed at 300 nL min^-1^. Peptides (5 µL) were initially trapped and washed on the columns at 3-µL min^-1^ for 20 minutes prior to eluting with the following gradient profile (min:%B): 0:5, 2:8, 20:12, 75:35, 97:60, 100:85. Data acquisition began 10 minutes after the start of the gradient and ended 10 minutes after the gradient end to account for column dead volume and allow the best gradient overlap with the two column system.

Data were acquired using a Velos Orbitrap mass spectrometer (Thermo Scientific) with a custom-made electrospray ionization (ESI) interface, using custom made, chemically-etched fused silica electrospray emitters (150 µm o.d. x 20 µm i.d. fused silica) (Kelly et al., 2006). The heated capillary temperature was set to 200ºC with a spray voltage of 2.2 kV. Data were acquired for a total of 100 minutes with a 10 minute delay from the start of the gradient. Orbitrap spectra (AGC 1x104) were collected from 400-2000 m/z at a resolution of 60k followed by data dependant CID MS/MS (collision energy 35%, AGC 1x106) of the six most abundant ions, excluding single charge states. A dynamic exclusion time of 30 sec was used to discriminate against previously analyzed ions using a 0.55 to 1.55 Da mass window.

**References**

Kelly, R.T., Page, J.S., Luo, Q., Moore, R.J., Orton, D.J., Tang, K., et al. (2006). Chemically etched open tubular and monolithic emitters for nanoelectrospray ionization mass spectrometry. *Anal Chem* 78(22)**,** 7796-7801.

Maiolica, A., Borsotti, D., and Rappsilber, J. (2005). Self-made frits for nanoscale columns in proteomics. *Proteomics* 5**,** 3847-3850.

Shevchenko, A., Tomas, H., Havli, J., Olsen, J.V., and Mann, M. (2007). In-gel digestion for mass spectrometric characterization of proteins and proteomes. *Nat Prot* 1**,** 2856-2860.

**Supplementary Figures**

**Figure S1. Unconjugated bilirubin interfered with Leucoberbelin blue (LBB) absorbance assay at 620 nm.** (A) Absorbance peaks at 450 nm (bilirubin) and 620 nm (LBB) could be identified when the LBB assay was run on test secretome samples from *P. sporulosum* (14 days) in the presence of bilirubin, as well as in secretome-free bilirubin controls. Note that secretome samples lowered absorbance at 450 nm compared to controls, indicating bilirubin oxidation. Mn(II) was not added here, and therefore the LBB peak at 620 nm is low. Bilirubin was prepared in 8.8% DMSO buffered by 20 mM HEPES pH 8.0. (B) Because absorbance peaks did not overlap in (A), we attempted to quantify Mn(IV) equivalents using the LBB assay on KMnO_4_ standards in the presence of 0-60 µM unconjugated bilirubin. The presence of bilirubin substantially lowered A620nm, particularly at low Mn concentrations, hindering Mn(IV) quantification in this range. Thus, Mn(II) oxidation in the presence of bilirubin was not measured for secretome samples in this study.

**
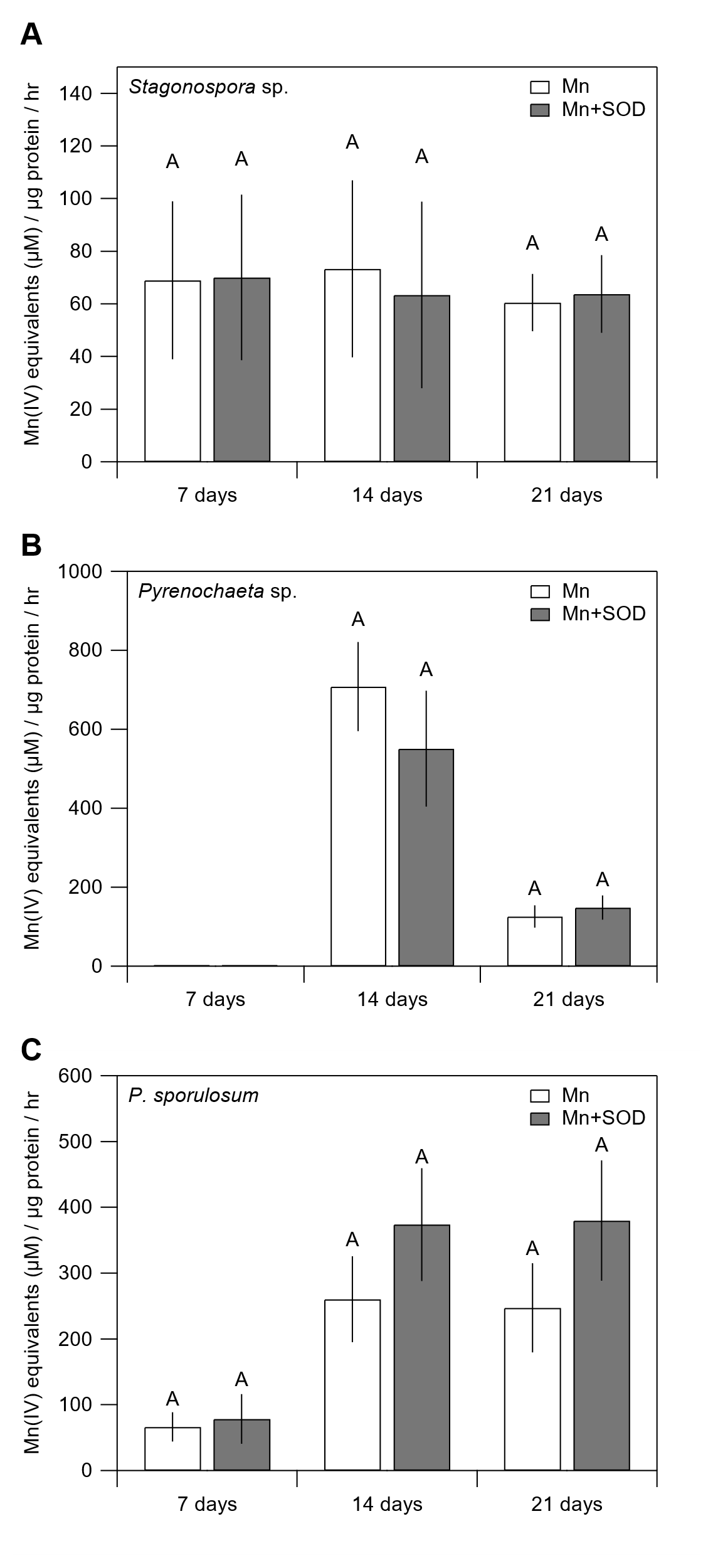
**

**Figure S2. Mn(II) oxidation rate was uninhibited by superoxide dismutase (SOD) in the secretomes of all 3 fungi.** Mn(II) oxidation rate in the untreated secretome (white bars) of (A) *Stagonospora* sp., (B) *Pyrenochaeta* sp., and (C) *P. sporulosum*, and after incubation with 50 kU L^-1^ SOD for 1 hour (gray bars). Samples were also incubated with 100-350 µM Mn(II) depending on initial Mn(II) oxidation rate (Figure 1). Data were analyzed with independent samples *t*-tests. Within each organism and time point, letters indicate statistically different groups. Error bars represent +/- 1 standard deviation (N=3-4 for each group).

| **GenBank accession number** | **Sequence shorthand** | **Genome** | **Protein description** |
| --- | --- | --- | --- |
| **OAL06359.1** | **Stago_tyrosinase** | ***Stagonospora* sp. SRC1lsM3a** | **Tyrosinase; di-copper centre-containing protein** |
| CAC82195.1 | Tyrosinase_Asco | *Aspergillus fumigatus* | Tyrosinase |
| PBP18673.1 | Tyrosinase_Asco | *Diplocarpon rosae* | Tyrosinase |
| EAA35587.2 | Tyrosinase_Asco | *Neurospora crassa* OR74A | Tyrosinase |
| XP_031001670.1 | PPO_Asco | *Lachnellula hyalina* | Polyphenol oxidase |
| OAT05486.1 | PPO_Asco | *Blastomyces gilchristii* SLH14081 | Polyphenol oxidase |
| GFP57945.1 | PPO_Asco | *Trichoderma asperellum* | Polyphenol oxidase 1 |
| **OAL02734.1** | **Stago_bilirubin** | ***Stagonospora* sp. SRC1lsM3a** | **Multicopper oxidase; cupredoxin** |
| **XP_018030874.1** | **Para_bilirubin** | ***P. sporulosum* AP3s5-JAC2a *(Paraphaeosphaeria sporulosa)*** | **Multicopper oxidase; cupredoxin** |
| RYN74507.1 | Bilirubin_Asco | *Alternaria alternata* | Bilirubin oxidase |
| EAA27114.3 | Bilirubin_Asco | *Neurospora crassa* OR74A | Bilirubin oxidase |
| RMI99061.1 | Bilirubin_Asco | *Fusarium kuroshium* | Bilirubin oxidase |
| GAQ36284.1 | Laccase_Asco | *Aspergillus niger* | Laccase |
| AAA33591.1 | Laccase_Asco | *Neurospora crassa* | Laccase |
| OAG18414.1 | Laccase_Asco | *Alternaria alternata* | Laccase |
| PTB67553.1 | MCO_Asco | *Trichoderma citrinoviride* | Multicopper oxidase |
| KJK64339.1 | MCO_Asco | *Aspergillus parasiticus* SU-1 | Multicopper oxidase |
| CAA70509.1 | MCO_Asco | *Candida albicans* | Multicopper oxidase |
| BAA24288.1 | Ascorbate_oxid Asco | *Acremonium* sp. HI-25 | Ascorbate oxidase |
| KAF1967560.1 | Ascorbate_oxid_Asco | *Bimuria novae-zelandiae* CBS 107.79 | Ascorbate oxidase |
| KYK60778.1 | Ascorbate_oxid_Asco | *Drechmeria coniospora* | Ascorbate oxidase |

**Figure S3.** Multiple sequence alignment of putative tyrosinase identified in *Stagonospora* sp. and putative bilirubin oxidases identified in *Stagonospora* sp. and *P. sporulosum* with known Ascomycota multicopper oxidase sequences obtained from GenBank. Sequences were aligned with CLUSTAL Omega version 1.2.4. Sequence descriptions are provided in the table, in which sequences from fungi in this study are shown in **bold**. Sequence alignment and consistency of conserved Cu-binding domains support the identification of the fungal proteins in this study as tyrosinase and bilirubin oxidase.

**Sequence alignment legend:**

**Tyrosinases & polyphenol oxidases (PPOs) (2-Cu MCOs):**

6 histidines associated with the conserved common central domain of tyrosinase (Pfam00264) are shown in **red**. This domain binds 2 Cu ions via 2 sets of 3 histidines. Common amino acid sequences in the immediate vicinity of these histidines are shown in **yellow**.

**Bilirubin oxidases (4-Cu MCOs):**

10 histidines associated with 3 conserved Cu-binding domains of bilirubin oxidases (CuRO_1_BOD, CuRO_2_BOD, and CuRO_3_BOD) are shown in **blue**. Common amino acid sequences in the immediate vicinity of these histidines are shown in **yellow**.

Conserved domains were identified using NCBI BLAST.

OAL06359.1_Stago_tyrosinase ------------------MKTFVSASLAI--VVGVSALPQITPSTPLAPALP-LQAFDTQ 39

CAC82195.1_Tyrosinase_Asco ------------------------------------------------------------ 0

GFP57945.1_PPO_Asco ------------------MLLQ-ASVWAL--ALATV------------------------ 15

EAA35587.2_Tyrosinase_Asco ---------------------MPMARFRL--PLALLFLAVFVAHFVLAQYGAYNYGFDAA 37

OAT05486.1_PPO_Asco ------------------MGPF---RWKV--LLLLN------------------------ 13

PBP18673.1_Tyrosinase_Asco ------------------MLPQIIAAWTV--LVALLFVD----ASPTAPSD--------- 27

XP_031001670.1_PPO_Asco ------------------MQLQ---SFCS--I-------------ALATFA--------- 15

RMI99061.1_Bilirubin_Asco ---------------------MKVTSFIL-PSLALNSLGGLCA----------AQ----- 23

OAL02734.1_Stago_bilirubin -----------------------MLSFFTQATLAL-LLSRPALA----QNNPSSN----- 27

XP_018030874.1_Para_bilirubin -----------------------MLSKLVRATLAL-TLTSSCV---------VQA----- 22

RYN74507.1_Bilirubin_Asco -------------------------------MKNYFSIGVAAL----------------- 12

EAA27114.3_Bilirubin_Asco -----------------------MASFSLSQTLLFLVLVLSAV----------------- 20

KAF1967560.1_Ascorbate_oxid_Asco ------------------------------------------------------------ 0

BAA24288.1_Ascorbate_oxid_Asco ------------------------------------------------------------ 0

KYK60778.1_Ascorbate_oxid_Asco ------------------------------------------------------------ 0

GAQ36284.1_Laccase_Asco MKEQGSILLDSQQSPSFRLRRLPWGIVLYPATICGLILLLFVAQ----ISQPSPL----- 51

CAA70509.1_MCO_Asco ------------------------------------------------------------ 0

PTB67553.1_MCO_Asco ------------------------------------------------------------ 0

KJK64339.1_MCO_Asco ------------------------------------------------------------ 0

AAA33591.1_Laccase_Asco -----------------------MKFLGIAALVAGLLAPPLVLA----APAPGTE----- 28

OAG18414.1_Laccase_Asco ---------------------MVASMFKVA-TALGLFL-PFVAS----LTVPS------- 26

OAL06359.1_Stago_tyrosinase KFT--TLLSL------DDILN-------GRFPKPSE-IIADNQTEEASF--ST-----LA 76

CAC82195.1_Tyrosinase_Asco -------MSSNKP----YVIKGIPVDAGQIIPVRRD--IDEWYEDTSRQ----------- 36

GFP57945.1_PPO_Asco -----SVAQGTAHIP----VTGVPVSSGSAVPLRQN--INDLVKSGPQW----------- 53

EAA35587.2_Tyrosinase_Asco KLIKRQLASQEPVPVVTGAEG------GETIRPRQE--IRQLEQDKELW----------- 78

OAT05486.1_PPO_Asco -----VLLGVASAVTISGARGGVNHH-TGERPARKD--INRLQWSGPEW----------- 54

PBP18673.1_Tyrosinase_Asco -----SISPHQRVHKIKGLHTGIKRQAGSPRPARRN--ILDLQKDGPAWHVSS-----FP 75

XP_031001670.1_PPO_Asco -----CIEIVNAGFPITGVQTGMQNGS-GPAPPRQN--ILDLYNDQI------------- 54

RMI99061.1_Bilirubin_Asco -----------------NWTY--------DLPPEESAALETVVEDDPNDVVYLMKS-DE- 56

OAL02734.1_Stago_bilirubin -----------------RFAS--------EVPQSETVALSNIVEDDPANLPAARKGGDE- 61

XP_018030874.1_Para_bilirubin -----------------QFVT--------QIPAAEASAIAGIVEDDPANDPQFRKIG--- 54

RYN74507.1_Bilirubin_Asco -------------------------------------LAS---------QALAGDDAWI- 25

EAA27114.3_Bilirubin_Asco -------------------------------------QLG---------SVSAGIDKWL- 33

KAF1967560.1_Ascorbate_oxid_Asco ------------------------------------------------------------ 0

BAA24288.1_Ascorbate_oxid_Asco ----------------------------------------------------------ML 2

KYK60778.1_Ascorbate_oxid_Asco -----------------------------------------------------------M 1

GAQ36284.1_Laccase_Asco -------------------QR--------LIPSRVST------------SQ-------IH 65

CAA70509.1_MCO_Asco ------------------------------------------------------------ 0

PTB67553.1_MCO_Asco ------------------------------------------------------------ 0

KJK64339.1_MCO_Asco ------------------------------------------------------------ 0

AAA33591.1_Laccase_Asco -----------------GVNL--------LTPVDKRQ-----------DSQAERYGGGGG 52

OAG18414.1_Laccase_Asco ------------------VEY--------LTPRVPEVPWK----ETGLWSGHQKRDGYKT 56

OAL06359.1_Stago_tyrosinase EDDTVSTMATCSN-V---RVRQEWDSYSDSDRQAFVSSVRCLMQRGPNGQF-SQSKSRYE 131

CAC82195.1_Tyrosinase_Asco ------------SRIQLSIFIWALREFQSIDYKDRLSYFQ---IAGIHHF----PLITWD 77

GFP57945.1_PPO_Asco -----------------DLYVQAMYNMSKLDSHDPYSFFQ---IAGIHGA----PYIEYN 89

EAA35587.2_Tyrosinase_Asco -----------------TLYILGLSLMQFTDQSSPVSWYG---ITGIHGI----PHQTWG 114

OAT05486.1_PPO_Asco -----------------DLYIQALKAYQEEDRSHLLSYHQ---VAGIHGY----PYKSWD 90

PBP18673.1_Tyrosinase_Asco RMPPTFDMFPCLGNSTPSLYIQGLSALQSKNESYFLSYFQ---IAGIHGRKMTSPYIPWG 132

XP_031001670.1_PPO_Asco ---------------QFSLYIQALNSFQQVNESNLTSYFQ---VAGIHGR----PYIAWD 92

RMI99061.1_Bilirubin_Asco -SPLYPLIYRNALPIPPVKKP--LKIIKNPVTGKDIWYF----------E----IEIKSF 99

OAL02734.1_Stago_bilirubin -SPAY-TLYSAALPIPPVAQV--KQKVINPVTNKEIWYY----------E----LDIKPF 103

XP_018030874.1_Para_bilirubin -SPAY-TLYSAALPIPEVATP--KYKITNPVTQKEIWYY----------E----FEIKPF 96

RYN74507.1_Bilirubin_Asco -SPVYKEIFQNELPIPKDKVK--SYTYTNKTTGNQIDFY----------E----VDVTPF 68

EAA27114.3_Bilirubin_Asco -SPPYKWTFQFPLPIPPVKAP--SKTIVNPITGKNILYY----------E----VEIKEF 76

KAF1967560.1_Ascorbate_oxid_Asco ------------------------------MSSKPTLHDANF---QPDFI----LRVTEE 23

BAA24288.1_Ascorbate_oxid_Asco LGTLFTLLAQCLLI---------------EATSCLVKHDGGF---VPDHV----LRVSSR 40

KYK60778.1_Ascorbate_oxid_Asco RRPLLLLLAHCLLA---------------T-AASLVTHGGQF---VPDHV----LRVSSA 38

GAQ36284.1_Laccase_Asco PTVHEPDVSRPLIE------L--HPEDHIYRNPSTQHHD---------------WVVTAD 102

CAA70509.1_MCO_Asco --------MRTFLS-SFIILT--TF-LASLIAAETHTWY---------------FKTGWV 33

PTB67553.1_MCO_Asco ----MNSLTRLLL-------A---A-SLGVASAATVKYD---------------FNITWV 30

KJK64339.1_MCO_Asco ----MAILAQILLI-SFAAFL---L-LFQSCRAKTVTYD---------------FNVTWV 36

AAA33591.1_Laccase_Asco GGCNSPTNRQCWSP-GFNINT--DYELGTPNTGKTRRYK---------------LTLTET 94

OAG18414.1_Laccase_Asco NCNHGPTSRGCWNG-DFDIDT--DMDLHWPNTGKTVKYH---------------LTITNG 98

OAL06359.1_Stago_tyrosinase DL-------VA------LHQTLTPNVHGNSKFLLWHRYFLWTFEQLLRDECGFD------ 172

CAC82195.1_Tyrosinase_Asco EE-------EPP-----VPNKPGYCVHNNVTFPTWHRPYMLLFEQRLFEIMETTI----- 120

GFP57945.1_PPO_Asco KA-------GAQT----GDGWLGYCPHGQALVSV-------------------------- 112

EAA35587.2_Tyrosinase_Asco GV-------TPTP----GNEETGYCTHSSILFPTWHRPYLALYEQVLYNLIQ-------- 155

OAT05486.1_PPO_Asco GV-------EGL-------GGAGYCSHGSTLFPVWHRPYLAMYEERISRYAH-------- 128

PBP18673.1_Tyrosinase_Asco GA-------AQAP----NASLTGYCTHNSVLFLPWHRPYLALYEEMIGALIQ-------- 173

XP_031001670.1_PPO_Asco DI-------GSDPTGTQNGIPSGYCPHGNTLFPTWHRPYLALYEASNSSSLCTSFLT--I 143

RMI99061.1_Bilirubin_Asco TKSVYP-NLRDATFTGYDGMAPGPTIMVPKGTESIVRFINNAAHE-NSVHLHGSYSRAP- 156

OAL02734.1_Stago_bilirubin THQIYP-GKKAARLVGYNGTSPGPTIIVPRGTETVVRFVNHGDRE-SSIHLHGSPSRAP- 160

XP_018030874.1_Para_bilirubin TQQVYP-KLKPARLVGYDGKSPGPTIIIPKGTESVVRFVNNADRE-NSVHLHGSPSRAP- 153

RYN74507.1_Bilirubin_Asco TQQVYR-GLKPARLVGYDGVSPGPTFRMKKGREAIVRFKNHGDKD-LSVHLHGSYSRAP- 125

EAA27114.3_Bilirubin_Asco KSQVYP-DRGPATLWGYDGMSPGPTFIVEKGTETVVRFVNNARLA-NSVHLHGSYSRAP- 133

KAF1967560.1_Ascorbate_oxid_Asco DVPFA--CEGLRKSVVINGTSLGPELRLRPNSTTWVRVYNDMDHLNTTIHWHGLAQRVAP 81

BAA24288.1_Ascorbate_oxid_Asco NISIA--CTS-RQSAVVNGTSPGPELRVPAGQRTWIRVYNDLEQENLTMHWHGLAQRMAI 97

KYK60778.1_Ascorbate_oxid_Asco DIAIA--CER-RRSAVVNGTVPGPTLRILAGRRTWIRVYNDMPKENLTMHWHGLAQRMAI 95

GAQ36284.1_Laccase_Asco H--RRPDGVLK-RVYLINDLFPGPTVEARSGDRLIVNVTNSLEEEPISIHWHGIHIE--N 157

CAA70509.1_MCO_Asco DA--NPDGVYPRKMIGFNDSWPLPTLRVKKGDRVQLYLINGFDNLNTTLHFHGLFVRGAN 91

PTB67553.1_MCO_Asco TA--NPDGAFARPVIGINDQWPIPRIEANVGDRIVINVNNQLGNQSTSLHFHGLYMNGTT 88

KJK64339.1_MCO_Asco TA--NPDGLHPRKVVGINGQWPLPVIEVDKGDRIVANVYNGLGDKDTSIHWHGIFQNTTN 94

AAA33591.1_Laccase_Asco DNWIGPDGVIKDKVMMVNDKIIGPTIQADWGDYIEITVINKLKSNGTSIHWHGMHQRNSN 154

OAG18414.1_Laccase_Asco TG--APDG-FERPILLINGQTPGPTILADWGDDLEITVTNDLQTNGTGLHWHGLRQLGSN 155

OAL06359.1_Stago_tyrosinase --RNLPWFD---------ETRYAGRF-AQSSIFSSQWLGSIN------------------ 202

CAC82195.1_Tyrosinase_Asco ----K-----ETVPE-SHKQEWRDAA-RQWRLPYWDFAKTSGPHATGPLSLPVLCGLANV 169

GFP57945.1_PPO_Asco AKKIANGY-----PA-QYRAQYQAAA-ASLRAPYWDWAADST---VPPCTVP----NTLK 158

EAA35587.2_Tyrosinase_Asco --NIAKWWP----EG-EPRNRYQAAA-LRFRIPYWDWASSPP---SGQSVLPLSVGGSPY 204

OAT05486.1_PPO_Asco --AIANTY-----PP-IIRGIYQKAA-SDLRIPYWDWASDP----ELPR-----SVITPE 170

PBP18673.1_Tyrosinase_Asco --DIVRTY-----PA-SMLSAYQAAA-NNFRIPYWDWASIP----TMPA-----VVNQPM 215

XP_031001670.1_PPO_Asco MQQVLAGFVQAAAKA-YNSTTYQTAA-DHFRIPFWDWAAVDQ---RFPD-----VMTWPS 193

RMI99061.1_Bilirubin_Asco -FDG---WAEDITQP-GEYKDYYYPN-GQSARLQWYHDHAMHLTAE-N----AYMGQAGA 205

OAL02734.1_Stago_bilirubin -FDG---WAEDLIMP-GQYKDYYYPN-GQSARFLWYHDHAVHFTAE-N----AYFGQAGA 209

XP_018030874.1_Para_bilirubin -FDG---WAADLTNR-GEYKDYYYPN-YQSARFLWYHDHAIHWTAE-N----AYFGQAGA 202

RYN74507.1_Bilirubin_Asco -FDG---WAEDTTKP-GQYKDYYYPN-KQSARTLWYHDHAIHHTAE-N----AYFGQAGF 174

EAA27114.3_Bilirubin_Asco -FDG---WAEDITPP-GWYKDYYYPN-SQSGRTLWYHDHAIDHTAE-N----AYYGQAGA 182

KAF1967560.1_Ascorbate_oxid_Asco FSDGTPLASQWPIPP-RYFFDYQLFVEPDDAGTYFYHAHIGM-----Q----AIT-AAGA 130

BAA24288.1_Ascorbate_oxid_Asco FADGSPQGSQWPIPP-GHFFDYELQTTVEDAGTYFYHSHVGM-----Q----ALT-ASGA 146

KYK60778.1_Ascorbate_oxid_Asco FSDGSPQGSQWPIPP-GHFFDYELHTTSDDAGTYFYHSHVGM-----Q----ALT-ASGP 144

GAQ36284.1_Laccase_Asco AMDGAVGVTQRAIPP-GSTFTYNFTIPTDQSGTFWYHAHSGL-----L----RADGLYGG 207

CAA70509.1_MCO_Asco QMDGPEMVTQCPIPP-GETYLYNFTV-TDQVGTYWYHSHTGG-----Q----YGDGMRGV 140

PTB67553.1_MCO_Asco HMDGPVGVSQCAIPP-GHSFTYDFTI--DQPGTYWYHSHHNA-----Q----YPDGLRGP 136

KJK64339.1_MCO_Asco NMDGPSMVTQCPIPP-GSSFTYNFTV--NQNGTYWYHCHTDA-----C----YPDGYRQA 142

AAA33591.1_Laccase_Asco IQDGVNGVTECPIPPRGGSKVYRWRA--TQYGTSWYHSHFSA-----Q----YGNGIVGP 203

OAG18414.1_Laccase_Asco EQDGVNGITECPIAP-GDSKVYKFKA--TQYGTTWYHSHYSV-----Q----YGDGLVGP 203

:

OAL06359.1_Stago_tyrosinase ---TGGDCVR------DG---------QFANLAINVG-----------PADRNELHCLAR 233

CAC82195.1_Tyrosinase_Asco VILNPANPET------PIELPNPVYKYRAPDLMGNLDKPFHIPPERIDPDKDDYYPWDKC 223

GFP57945.1_PPO_Asco INVPSGSGIR------TVDYTNPLRTFYFPHIALSGS-Y--------GDFSGGGQDHTIR 203

EAA35587.2_Tyrosinase_Asco VDVNGPNGVQ------RI--ANPLFSYSFK--PLNAT-AFLQDPW-------DIWTTTLR 246

OAT05486.1_PPO_Asco LNINTPEGLR------TV--RNPLYDYAIN--PSAQE-GFPID-------SLSRYHRTVR 212

PBP18673.1_Tyrosinase_Asco VQITTPSGLK------NV--TNPTFRYVFHEFPLNQS-YFPSDQSVEGDAWLSQYPYTVR 266

XP_031001670.1_PPO_Asco VSINTPNGTR------NV--TNPLYRYTFLNHPEPAN-WFPTDQE----TYLGSQPWTIR 240

RMI99061.1_Bilirubin_Asco YILTDSTE-DSL-GLP--------SGYGVYDIPLILSSKQYNEDGTVFS-------TVGE 248

OAL02734.1_Stago_bilirubin YLVTDPAD-NAL-GLP--------SGYGQFDIPLVLASKYYNSDGTLKS-------SLGE 252

XP_018030874.1_Para_bilirubin YLITDPEE-DQL-GLP--------SGYGKQDIPLVLSSKQYNSDGTLFS-------TQGE 245

RYN74507.1_Bilirubin_Asco YILHDPAE-DAL-GLP--------S--GAYDVPLALASKQYNSDGTLFD-------PKDE 215

EAA27114.3_Bilirubin_Asco YILHDPSIESPL-GLP--------SGYGTFDIPLILSAKQYTSSGSLFS-------PADE 226

KAF1967560.1_Ascorbate_oxid_Asco LIVEDNDEPP--------------YQYDD-ERTIIFSDFFNKTDDEMEQ-------GLL- 167

BAA24288.1_Ascorbate_oxid_Asco LIVEGCERPP--------------YQYDD-ERTLHWSDFFPQTDHEIEV-------GLQ- 183

KYK60778.1_Ascorbate_oxid_Asco LVVEDCRPPP--------------FRYDD-ERILHWSDYFRQDDAAFEA-------GLT- 181

GAQ36284.1_Laccase_Asco LIVHEPSPKSTVRGLLARADQQELGSYDK-DILLLVGDWYHRSADQVYS-------WY-M 258

CAA70509.1_MCO_Asco FIIEDDDFPY-------------H--YDE-EVVLTLSDHYHKYSGDIGP-------AFLT 177

PTB67553.1_MCO_Asco LIIHDPKFPY-------------RKEVDQ-ELVLTLSDWYHDQMATLLP-------IFLS 175

KJK64339.1_MCO_Asco LIVHDKDAYF-------------NDMYDD-EFTLTLSDWYHELVEDI---------TFIS 179

AAA33591.1_Laccase_Asco IVINGPASAN----------------YDVDLGPFPLTDYYYDTADRLVL-------LT-Q 239

OAG18414.1_Laccase_Asco MIIRGPATAN----------------YDIDLGVLPMTDWFHATTFTVNA-------AAVH 240

OAL06359.1_Stago_tyrosinase -----------N------NDDSKTINTGSQ-------------------------FVDGC 251

CAC82195.1_Tyrosinase_Asco QATTKYGLLKNNPHIQDAGQDVTKSNLALNEHPWYRPNKAGFPPLQTLTYEVHRLLSFKF 283

GFP57945.1_PPO_Asco -----------CPSPQENYPNTANAN-------------LQARPYKGWIYD----VLTNS 235

EAA35587.2_Tyrosinase_Asco -----------SPTTSDNKAQSNNSLVAVNFD-------QNLDSIGQRLYI----LFSNY 284

OAT05486.1_PPO_Asco -----------NPDE---QGVSQVEAIQRTLN-------ANGALIRINTYQ----LLAGE 247

PBP18673.1_Tyrosinase_Asco -----------GARS---HGDPSDPDLANSVL-------Q-SSNLKSSTWY----ALVKP 300

XP_031001670.1_PPO_Asco -----------QPDG---NNVSDEAAMESQFV-------QEGQFLSDQVWS----IFAKT 275

RMI99061.1_Bilirubin_Asco ---------KD--SL--------------------------------------------- 252

OAL02734.1_Stago_bilirubin ---------EE--SL--------------------------------------------- 256

XP_018030874.1_Para_bilirubin ---------TK--SL--------------------------------------------- 249

RYN74507.1_Bilirubin_Asco ---------TD--SL--------------------------------------------- 219

EAA27114.3_Bilirubin_Asco ---------TT--SL--------------------------------------------- 230

KAF1967560.1_Ascorbate_oxid_Asco ----------AVPFKWT------------------------------------------- 174

BAA24288.1_Ascorbate_oxid_Asco ----------SVPLVWP------------------------------------------- 190

KYK60778.1_Ascorbate_oxid_Asco ----------AVPFVFG------------------------------------------- 188

GAQ36284.1_Laccase_Asco ---------RAGSFGNE------------------------------------------- 266

CAA70509.1_MCO_Asco ---------RFNPTGAE------------------------------------------- 185

PTB67553.1_MCO_Asco ---------KNNPTGAE------------------------------------------- 183

KJK64339.1_MCO_Asco ---------VTNPTGAE------------------------------------------- 187

AAA33591.1_Laccase_Asco ---------HAG----P------------------------------------------- 243

OAG18414.1_Laccase_Asco ---------ANG----P------------------------------------------- 244

OAL06359.1_Stago_tyrosinase NNQGSYSEMASCS--------------------EGSAHAWGHN--G------------IG 277

CAC82195.1_Tyrosinase_Asco SSWGAFASTKWCNEENKPPASQQTRDILSLEYIHNNVHNWVGG--TDYLGDPSKPDLQGA 341

GFP57945.1_PPO_Asco QNFADFASTSG--------------PGINVEQIHNAIHWDGAC----------------G 265

EAA35587.2_Tyrosinase_Asco GNYSTFSNNAWIPY-------INNGSFDSLEAIHDTVHNLAGG--GGL-GQP----NAQG 330

OAT05486.1_PPO_Asco TNYTVFSTDALP---------DRHGSFNNLENIHGLIHNSVGG--N-------------G 283

PBP18673.1_Tyrosinase_Asco ITFNDFGTTAT--------------SGTSIEAPHNQVHGSIGM--G-------------G 331

XP_031001670.1_PPO_Asco RNYNNMSTTSN--------------KGNAFEGPHGTVHVLIG---G-------------D 305

RMI99061.1_Bilirubin_Asco --------------------------------WGDIIEVNGQ------------------ 262

OAL02734.1_Stago_bilirubin --------------------------------WGDVVHVNGV------------------ 266

XP_018030874.1_Para_bilirubin --------------------------------WGDVIHVNGQ------------------ 259

RYN74507.1_Bilirubin_Asco --------------------------------WGDVIHVNGQ------------------ 229

EAA27114.3_Bilirubin_Asco --------------------------------YGDVIHVNGQ------------------ 240

KAF1967560.1_Ascorbate_oxid_Asco -------------------------------GETNAVLLNGKGISKLRQGLNGTLDFNST 203

BAA24288.1_Ascorbate_oxid_Asco -------------------------------GEVRAVLLNGKGIGIGHEADVSPS----- 214

KYK60778.1_Ascorbate_oxid_Asco -------------------------------GESQGVLLNGKGVALGREGAEGPL----G 213

GAQ36284.1_Laccase_Asco -------------------------------PVPDSLLINGVGHFDCSMAVPARPVDCIL 295

CAA70509.1_MCO_Asco -------------------------------PIPQNFLFNET------------------ 196

PTB67553.1_MCO_Asco -------------------------------PVPKAALMNET------------------ 194

KJK64339.1_MCO_Asco -------------------------------PVPDSFLVNDT------------------ 198

AAA33591.1_Laccase_Asco -------------------------------PPSNNVLFNGFAKHPTTGAG--------- 263

OAG18414.1_Laccase_Asco -------------------------------PVADNVLINGTMT--SAAGG--------- 262

OAL06359.1_Stago_tyrosinase AVMQDTWASPADP----VFWLHHAFIDRNFRIWQNQNS----------------ARINNI 317

CAC82195.1_Tyrosinase_Asco GHMSSVPVAAFDP----IFWLYHNNVDRLTAIWQVLNQ----------------DHWFDE 381

GFP57945.1_PPO_Asco NQFLAPDYSGFDP----LFFMHHSNVDRMWAFWEAIMP----------------SSPVFT 305

EAA35587.2_Tyrosinase_Asco GHMAYIPYSSFDP----IFFLHHAMVDRIFAIWQSLYP----------------SSYVTP 370

OAT05486.1_PPO_Asco GHMTYTPWSAYDP----IFWLHHTNVDRVVALWQAVHP----------------ESYVQP 323

PBP18673.1_Tyrosinase_Asco GHMSVLSYSAYDP----IFWLHHANVDRLFALWQALNP----------------NAYLTP 371

XP_031001670.1_PPO_Asco GHMTYLSFSAFDP----AFFLHHANVDRQIAMWQAIYP----------------GEWLQP 345

RMI99061.1_Bilirubin_Asco ---AWPSLK-VEPR-KYRFRFLNAAISRSFALYFVNSN-ALNSRLGFQVISSDTGLLSKP 316

OAL02734.1_Stago_bilirubin ---PWPSMN-VQPR-KYRFRILNAAVSRNFDLYFVKST-ATNTKLPFKVIASDAGLLQNA 320

XP_018030874.1_Para_bilirubin ---PWPFHN-VEPR-KYRFRFLNAAVSRNFDLFFVTST-ATNTRIPFKVIASDAGLLEAP 313

RYN74507.1_Bilirubin_Asco ---PWPYMK-VEPR-KYRFRMLNTAISRAFKLTLEDP---TAKKVPFHVIGADTGLMTKP 281

EAA27114.3_Bilirubin_Asco ---PWPFLN-VEPR-KYRFRFLDASISRSFLLYFERDAKVGGTKLPFDVIASDAGLLNAP 295

KAF1967560.1_Ascorbate_oxid_Asco EICQLPVID-VDPGKTYRFRFIGSTALSHVLAGFEGHP-------DLEIINIE-GHYTKP 254

BAA24288.1_Ascorbate_oxid_Asco GDCSLPVID-VDPGKTYRFRFIGATGLSLVSMGFEGHQ-------NLTIIQVDGGEWTKP 266

KYK60778.1_Ascorbate_oxid_Asco SECSLPVID-VEPGRTYRFRFIGSTGLSLVSIAFEGHV-------NLHVVQADAGAWTRP 265

GAQ36284.1_Laccase_Asco RHMNVSYLD-AKGDAAYRVRVVNTGSVAGFTLGFQNRE--------FSLIQVD-NIDVEQ 345

CAA70509.1_MCO_Asco ---RNATWK-VEPGKTYFVRILNVGGFVSQYLWMEDHE--------FTIVEID-GVYVEK 243

PTB67553.1_MCO_Asco ---QNLTVS-VEPNTTYMFRVINIGAFAGQYLWIEGHT--------MRIVEVD-GVYTEQ 241

KJK64339.1_MCO_Asco ---QGSSLA-VEPGKTYLLRLINMGAFVGMYFYIEDHS--------FTIVEMD-GVYTDP 245

AAA33591.1_Laccase_Asco ---QYATVS-LTKGKKHRLRLINTSVENHFQLLLVNHS--------MTIISAD-LVPVQP 310

OAG18414.1_Laccase_Asco ---KYAETI-LTPGKTHLLRLVNTGINNYLHVGLDGHS--------FQVISAD-FTPIEP 309

. .

OAL06359.1_Stago_tyrosinase DGT--------------------------------------------------------- 320

CAC82195.1_Tyrosinase_Asco PHP--------------------------------------------------------- 384

GFP57945.1_PPO_Asco SSY---------------------K-----------------------------G-QSRF 314

EAA35587.2_Tyrosinase_Asco MQA---------------------------------------------------Y-IASY 378

OAT05486.1_PPO_Asco LPN---------------------------------------------------R-GGDF 331

PBP18673.1_Tyrosinase_Asco QID---------------------------------------------------Q-YGTF 379

XP_031001670.1_PPO_Asco EAA---------------------------------------------------A-SGTW 353

RMI99061.1_Bilirubin_Asco VQVSD-IYVSMAERYEIVFDFSKYAS-----QTLELRNLEKVG---GIGTDDDYENTDKV 367

OAL02734.1_Stago_bilirubin VQVQH-VITAVAERWEIVMDFSAYAG-----QTILLRNNQDAG---GIGTDDEYDNTDKV 371

XP_018030874.1_Para_bilirubin IQVSD-LKVAAAERYEVVFDFSQYAG-----KTLYLRNDEDAG---GIGVDEAYDNTNQV 364

RYN74507.1_Bilirubin_Asco VQSDN-LEISMAERWEIVVDFSTYSG-----KNVTMKNARD------VQADEDYNSTDKV 329

EAA27114.3_Bilirubin_Asco QRVNE-LYISMAERYEVVVDFGGYKG-----ENITLKNARD------VGADRDFPDTDKV 343

KAF1967560.1_Ascorbate_oxid_Asco YTVQH-MQLGSGERFDILFRTKSWAELLAE-GRDSYFIQFETR-----DRPESIT-GFGI 306

BAA24288.1_Ascorbate_oxid_Asco ASVDR-IQLASGQRFDALFKAKTEEELASE-GRQTYFIQFETR-----DRPEVYR-GYAV 318

KYK60778.1_Ascorbate_oxid_Asco VWVDR-IQLGSGQRFDAIFKAKTAEELRKAGGKMTYVVQYETR-----DRPSVYR-GYAV 318

GAQ36284.1_Laccase_Asco QDSNSAGVLYPGQRMDIILRPSPEKA------PSSLTIDLDKECFRYPNPA-----LTSV 394

CAA70509.1_MCO_Asco NTTDL-IYITVAQRYGVLITTKNSTD-----KNYVFMNGVDTTMLDSVPADLQVN-GTNY 296

PTB67553.1_MCO_Asco AEAEM-VYISAAQRVSFLLTTKNDTS-----VNFPIVASMDTSLFDTLPEDLNYN-VTGW 294

KJK64339.1_MCO_Asco TEADL-LYIAVAQRYSILVTTKNSTA-----KNYPIVTVVDSSLLDVIEPDLQLN-HTNW 298

AAA33591.1_Laccase_Asco YKVDS-LFLGVGQRYDVIIDANQAV------GNYWFNVTFGGSKLCG--DSDNHY-PAAI 360

OAG18414.1_Laccase_Asco FWTDN-LILSVGQRYEIIINATEPI------GNYWWRVGTGGRCDGP--NANAAN-IKSI 359

OAL06359.1_Stago_tyrosinase -DKAGNPLTLDTTVNVYGFRPDVRIRDILDTTATTLCYKYNY------------------ 361

CAC82195.1_Tyrosinase_Asco -----SDAKPDDPLKPFHVSKD-KYF-TSDDARFWRKYGYDYDIVKKPGTNE-D------ 430

GFP57945.1_PPO_Asco NSKTGATITPNSPLQPFFQ-ANGQFH-TSNSVKSIQGMGYSYQGIEYWQKSQA------- 365

EAA35587.2_Tyrosinase_Asco TTSRGEFQTAATPLTPFYFNANGTFW-TSDMVRDHTRFGYTYPELVGVSGSVPNSQSRAL 437

OAT05486.1_PPO_Asco MKRPGTWEDAKTPFAPFRDPNN-RFY-NAETSRSMRPFGYTYPEIRDWGVTP-E------ 382

PBP18673.1_Tyrosinase_Asco SIAANSLDTASTPLEPFAANGDSPYF-TSSSVRQTSTFGYTYPEIQDWNQSP-A------ 431

XP_031001670.1_PPO_Asco TIYPNTQIDETTPLAPFTSSDGKTLY-TPATSRYTQNFGYSYNDAPYWQLPNSS------ 406

RMI99061.1_Bilirubin_Asco MRFVVS---------------S--------DKVE--D----NSTVPEKLRDISFARPE-- 396

OAL02734.1_Stago_bilirubin MQFKVS---------------A--------TPVT--D----TSTVPQNLRTVPFPPAS-- 400

XP_018030874.1_Para_bilirubin MKFVVS---------------S--------TPVT--D----TSVVPSTLRTVPFPPAG-- 393

RYN74507.1_Bilirubin_Asco MRFVVG---------------T--------SVSDTAG----NGALPGSLRTVPFPPKK-- 360

EAA27114.3_Bilirubin_Asco MRFVVS---------------D--------VAVVDDE----SSRVPAVLRSVPFPKTK-- 374

KAF1967560.1_Ascorbate_oxid_Asco IRYNPMAIPPTPPSSGVITLPK--------DIYDWAE----YSLEPLYPDYQPCPSAS-- 352

BAA24288.1_Ascorbate_oxid_Asco IRYSKASTTPHVPTIPPLTLPN--------NTYDWLE----YELRPLIET-VTQPTLG-- 363

KYK60778.1_Ascorbate_oxid_Asco LRYANGGDFPGRPATPPLRIPE--------KTNAWLE----YQLQPLYPGPRGHPTLA-- 364

GAQ36284.1_Laccase_Asco QTYNI--KKSPNNLAPTISPSN--------NTISLSE----VATRKSLLSGLPANSHQ-- 438

CAA70509.1_MCO_Asco IVYNE-SSALPDAYDIDSY------------DDALDD----FYLKPLSKQKLMDDADY-- 337

PTB67553.1_MCO_Asco LTYDS-QATLPEPAL-----VD--------ELDPFDD----MDLVPYDKMELLPEPDQ-- 334

KJK64339.1_MCO_Asco LEYNS-TADHPQAVMTVSD-SS--------DLVPFDD----ITLVPHDRTPLFQDPDM-- 342

AAA33591.1_Laccase_Asco FRYQGAPKALPTN--QGVA-PV--------DHQCLDL----NDLKPVLQRSLNTN----- 400

OAG18414.1_Laccase_Asco FRYAGGPAGEPNS--TA-R-VL--------PVGCYDE----QNIVPYAKTTVPQEMPE-- 401

OAL06359.1_Stago_tyrosinase ------------------------------------------------------------ 361

CAC82195.1_Tyrosinase_Asco --RAPEEVKMKINQLYGEPISRLH------------------------------------ 452

GFP57945.1_PPO_Asco --QIKSSVTTIINQLYGPKSSSKRNTRAAD---LVQT----------------------- 397

EAA35587.2_Tyrosinase_Asco SRRARGRVMAAINRLYGPSTPSSLYRKELRAGRLGPG------KKVPS------------ 479

OAT05486.1_PPO_Asco --QLSRNVRMEINRLYNRPGDNNRKRSTALTARAAHHHGMIMHKRMPNLAEDTLDDLKKL 440

PBP18673.1_Tyrosinase_Asco --QLTSNVTAAITKLYSPNGSRVN-------RRAGDTQ---------------------- 460

XP_031001670.1_PPO_Asco --DLSANVTARVNQLYNGDGHLGTWPA----KRSIGVG---------------------- 438

RMI99061.1_Bilirubin_Asco --TNEINHHFR------FHRTNGEWRI----NGVGFAD-----AANR------------- 426

OAL02734.1_Stago_bilirubin ---TGIDHEFR------FHRSNSQWQI----NDVGFAD-----AANR------------- 429

XP_018030874.1_Para_bilirubin --HTSIDHAFR------FHRSGGEWKI----NDVGFAD-----AANR------------- 423

RYN74507.1_Bilirubin_Asco ---SGIDRSFK------FGRTNGQWTV----NGVTFAD-----VNNR------------- 389

EAA27114.3_Bilirubin_Asco ---TQVDQHFK------FERSNGEWKI----NGVSFAD-----VANR------------- 403

KAF1967560.1_Ascorbate_oxid_Asco --EVTRRIVLNTTQMNDI-SRRIIWSF----NNLTWSE-----ATFQSPL--LVDVYKRG 398

BAA24288.1_Ascorbate_oxid_Asco --EVTRRVIINASQLTDPQNQHVVWRL----ANLSWTE-----AVRQTPL--LVDIYKFG 410

KYK60778.1_Ascorbate_oxid_Asco --EVTRRVVMNSSQLVDPRTGQVVWRL----AGLSWTD-----NAARTPL--LVDIYKRG 411

GAQ36284.1_Laccase_Asco --TYVVY----TKIEKLSINHNVPYGF---FNRTSWRP------QIDTPL----IDLPRE 479

CAA70509.1_MCO_Asco --TITVDVQMN------VLNDGINYAF---FNNISYKA-----PKVPTLL----TVLSAG 377

PTB67553.1_MCO_Asco --VVELDVIMD------NLRDGKNYAF---FNNITYTH-----PKVPSLY----TALSAG 374

KJK64339.1_MCO_Asco --VIELTVIMD------NLANGAGYAF---FNNISYTK-----PKVPTLY----SVLSSG 382

AAA33591.1_Laccase_Asco ----SIALN--TGNTIPITLDGFVWRVNGTAININWNK-----P---VLE---------- 436

OAG18414.1_Laccase_Asco --SLTFGFNDNYTSDVTQSQGLVQWLVNGNPMAIDLDR-----P---TLQ---------- 441

OAL06359.1_Stago_tyrosinase ------------------------------------------------------------ 361

CAC82195.1_Tyrosinase_Asco ---------------------------------EGQPVEYDYVINVIYDRY--------A 471

GFP57945.1_PPO_Asco ----------------------------------------RYFAQISVNVT--------D 409

EAA35587.2_Tyrosinase_Asco N--------------L-----------PVGRVFSGNGQYREWLANVRVKKQ--------A 506

OAT05486.1_PPO_Asco NFKVKEFFEDLGKFSL-----------LNFIKLGINNLEKQWVINIRANKF--------A 481

PBP18673.1_Tyrosinase_Asco -----------------------------STGLLPGQQTREWSVGIRVAKF--------D 483

XP_031001670.1_PPO_Asco -----------------------------GSGLEKRTVDRDWSVAVQVPNA--------A 461

RMI99061.1_Bilirubin_Asco ---------------------------VLAKVPRGT-------VEIWELEN------SSG 446

OAL02734.1_Stago_bilirubin ---------------------------VLAKVPRGK-------VEIWKLEN------KSG 449

XP_018030874.1_Para_bilirubin ---------------------------VLAKVPRGK-------VEIWELEN------NSG 443

RYN74507.1_Bilirubin_Asco ---------------------------ILAKPQRGA-------VEVWELEN------SSG 409

EAA27114.3_Bilirubin_Asco ---------------------------VLARPKRGT-------VEVWELEN------SSG 423

KAF1967560.1_Ascorbate_oxid_Asco EAALPSYSIATSEFQLNATYPQGYDPNVKAFPVRNG----EV-IEIIFQNTGSLVNFNGG 453

BAA24288.1_Ascorbate_oxid_Asco DLAIPNYDAALA--------NYGWDPETRAFPAKVG----EV-LEIVFQNTGSLVGSDGA 457

KYK60778.1_Ascorbate_oxid_Asco DAAMPSYDDAVR--------NGGWDPRTKAFPVKVG----EV-IEIVLQNTGSLK-NGGS 457

GAQ36284.1_Laccase_Asco EWDENQ-----------LVLSTG---STTSRPLSSEH-DQDLWIDLVVNNL--------D 516

CAA70509.1_MCO_Asco EAATNE-----------LIYGT----NTNSFVLQGG----DI-VDIVLNNF--------D 409

PTB67553.1_MCO_Asco DLADNA-----------AVYGE----YTHPFVLNKG----EI-VQIVVNNL--------D 406

KJK64339.1_MCO_Asco YLATNP-----------TVYGE----YTHPMVLERN----QI-VEIVLNNG--------D 414

AAA33591.1_Laccase_Asco YVLTGN-----------TNYSQ----SDNIVQVEGV----NQ-WKYWLIEND-PD--GAF 473

OAG18414.1_Laccase_Asco DVLDRN-----------VTYGN----NRHVFEIDEN----HK-WQYWVIQQD-TTNNTAP 480

OAL06359.1_Stago_tyrosinase ------------------------------------------------------------ 361

CAC82195.1_Tyrosinase_Asco LDGIPYTIVFYLHLKDG-------SYKCLGGVYTFST---------KLSDAQD----TER 511

GFP57945.1_PPO_Asco IPIRPAEINVYVAG------------QKAGSLIVMKL---------PAE----------- 437

EAA35587.2_Tyrosinase_Asco LD-GPFFIHLFLGEAPKDPKEWASAKNHVGSMGVFAS---------DERYGES----KMD 552

OAT05486.1_PPO_Asco LP-HAYNIHFFLSEPPEESCDWASAPNLIGTFASFAS---------SMGSPAN------- 524

PBP18673.1_Tyrosinase_Asco LDGERFIVRLFLGAIPRDPGAWATSPSCVGSFPVFPP---------PTPA-TG------- 526

XP_031001670.1_PPO_Asco VS-EPFSVGVTVGS------------TLVGKMFILHT---------PSQIELD------- 492

RMI99061.1_Bilirubin_Asco GWTHPIHVHLVDFLVLD---------RE-GRRGTMPY-------------E--------- 474

OAL02734.1_Stago_bilirubin GWTHPVHVHLVDFRILQ---------RS-G-RGVEPY-------------E--------- 476

XP_018030874.1_Para_bilirubin GWTHPIHVHLVDFRVIQ---------RN-G-RGVEPY-------------E--------- 470

RYN74507.1_Bilirubin_Asco GWSHPVHIHLVDFQILT---------RTGGKRSVLNY-------------E--------- 438

EAA27114.3_Bilirubin_Asco GWTHPIHIHLVDFRVKK---------RVNGKRSVLPY-------------E--------- 452

KAF1967560.1_Ascorbate_oxid_Asco VDVHPFHIHGKHYCDIG---------SCERGIYMS----YLVHSSGPGAYDWEENE--KK 498

BAA24288.1_Ascorbate_oxid_Asco VDIHPFHAHGEHFYDIG---------S------------------GDGVYDAEANE--AK 488

KYK60778.1_Ascorbate_oxid_Asco VDVHPFHAHGQHFYDLG---------S------------------GDGLYDAAANE--AK 488

GAQ36284.1_Laccase_Asco DSGHPFHMHGHHFYILR---------TYQAPVGWGAYNPFTD-AHPPGLALSSGSSSKAD 566

CAA70509.1_MCO_Asco TGKHPFHLHGHVFQLIE---------RHEAIG---------S-KESAVTFNVSDHA---- 446

PTB67553.1_MCO_Asco SGRHPFHLHGHAFQAIH---------RSEEEAGTF--ED-----------EALSES---- 440

KJK64339.1_MCO_Asco TGSHPFHLHGHNFQVIT---------RYPSYADGF--FSYAD-SDDPVTYDSANHS---- 458

AAA33591.1_Laccase_Asco SLPHPIHLHGHDFLILG---------RSPDVTAIS--QTRYV-FDPAV----DMAR---- 513

OAG18414.1_Laccase_Asco PLPHPIHLHGHDFYVLD---------QQENAV-----------W--NG----DISR---- 510

OAL06359.1_Stago_tyrosinase ------------------------------------------------------------ 361

CAC82195.1_Tyrosinase_Asco GGCDNCREQKKAGVLASA----------QIPLTYTLY----------------------- 538

GFP57945.1_PPO_Asco -------------GTVNG----------GFTVDNPMRTL----L--HGG----------- 457

EAA35587.2_Tyrosinase_Asco GM---------DEVMVSG----------TVPLTKALVEK----V--MGTGRFGVGGGVKG 587

OAT05486.1_PPO_Asco RT-----------RDMYG----------QIPLSHVLAVV----W---------------- 543

PBP18673.1_Tyrosinase_Asco PL---------PQVIAYS----------EVSLVQALQ----------------------- 544

XP_031001670.1_PPO_Asco AG---------VDRCTHA----------EFTLKNALS----------------------- 510

RMI99061.1_Bilirubin_Asco ------SAGLKDVVWL---------GKGETVRVAAVYAPFDGLYMFHCHNLIHEDSD--- 516

OAL02734.1_Stago_bilirubin ------AAGLKDVVWL---------GKNEEVLVEAHYAPWNGVYMFHCHNLIHEDHD--- 518

XP_018030874.1_Para_bilirubin ------SAGLKDVVWL---------GVNEVVLVEAHYAPWNGVYMFHCHNLIHEDQDASS 515

RYN74507.1_Bilirubin_Asco ------KEALKDVVLL---------GVNEKVTVIARYAPYDGVYMFHCHNLIHEDHD--- 480

EAA27114.3_Bilirubin_Asco ------AQGLKDVVWL---------GPGETVTVEAHYAPWDGVYMFHCHNLIHEDHE--- 494

KAF1967560.1_Ascorbate_oxid_Asco IASLNWTPIQRDTAMLYRYETRTTAGAPAGWRAVRIKMDQPGVWMVHCHVLQHMVMG--- 555

BAA24288.1_Ascorbate_oxid_Asco LVAMNYTAVKRDTTMLYHYAATTTPGAPAGWRAWRLRVTQPGVWMIHCHILQHMVMG--- 545

KYK60778.1_Ascorbate_oxid_Asco VVAMGYKAVERDTTMLHRYSNKTEPGAPAGWRVWRLRVEQPGVWLLHCHTLQHMMMG--- 545

GAQ36284.1_Laccase_Asco SPYDLSRAQLRDTVYI----------PSRGHAVLRFRADNPGIWLFHCHIIWHQASG--- 613

CAA70509.1_MCO_Asco --EWPEYPMIRDTVYV----------KPHSYMVLRFKADNPVVWFFHCHVDWHLEQG--- 491

PTB67553.1_MCO_Asco --DYKAVPMRRDTLVI----------WPNGNIVMRFKADNPGVWLFHCHIEWHVASG--- 485

KJK64339.1_MCO_Asco --SFPTYPARRDTFVV----------PPQGYFVARFVANNPGVWLFHCHIDWHLMQG--- 503

AAA33591.1_Laccase_Asco --LNGNNPTRRDTAML----------PAKGWLLIAFRTDNPGSWLMHCHIAWHVSGG--- 558

OAG18414.1_Laccase_Asco --LKTNNPIRRDTANL----------PARGYLVLAFESDNPGAWLMHCHIPFHVAAG--- 555

OAL06359.1_Stago_tyrosinase ------------------------------------------------------------ 361

CAC82195.1_Tyrosinase_Asco ------------------------------------------------------------ 538

GFP57945.1_PPO_Asco ------------------------------------------------------------ 457

EAA35587.2_Tyrosinase_Asco IAASAGGRS-----------------V--------------GGRFVGFEGD-VGNG---- 611

OAT05486.1_PPO_Asco ------------------------------------------------------------ 543

PBP18673.1_Tyrosinase_Asco ------------------------------------------------------------ 544

XP_031001670.1_PPO_Asco ------------------------------------------------------------ 510

RMI99061.1_Bilirubin_Asco MMAAFNVTSLDDFGYNS-TQF--LDPMQKEWRAKPYELSDVKGRTGPFSDEAIVETVESM 573

OAL02734.1_Stago_bilirubin MMAAFNVTQLQELGYNEQTDF--SDPEDPRWSAVPFVQADWTSRSGPFTSQAVAARIREI 576

XP_018030874.1_Para_bilirubin MMAAFNATQLTDFGYDEVTDY--GDPEDPRWSARPYVYTDFQQGTGPFSEQSVTDRVQEI 573

RYN74507.1_Bilirubin_Asco MMAAFNVTSLADFGYPETTKF--IDPMEEKYRSKDIND-------HDNEEEHIYEKCAEL 531

EAA27114.3_Bilirubin_Asco MMAAFNVSVLQDLGYDE-THY--IDPMEARWRARQQSV-------EAFDDSAVEARVKEM 544

KAF1967560.1_Ascorbate_oxid_Asco MQMVWTVGSAEDILKIPTELA---------------------AGYLEYGGSSYGNETYSP 594

BAA24288.1_Ascorbate_oxid_Asco KSADAV------------------------------------------------------ 551

KYK60778.1_Ascorbate_oxid_Asco MQSAWVVGSADQIRNIPFHYA---------------------QGYLEYGGDAYGNSTHEP 584

GAQ36284.1_Laccase_Asco MAMLLQI----------------------------------------------------- 620

CAA70509.1_MCO_Asco LAVVLIEDPQAIQKNEKITEN--HKRICEK-------------VGVPWEGNAAANSND-- 534

PTB67553.1_MCO_Asco LLATFVEAPLDIQKQFTIPDD--HLAVCDA-------------GSVPTKGNAAGNAAD-- 528

KJK64339.1_MCO_Asco LAMVFIEAPFEIQQRTTVPDD--HWAACAA-------------GNVSDKGNAAGNTED-- 546

AAA33591.1_Laccase_Asco LSNQFLERAQDLRNSISPADKKAFNDNCDAWRAYF-------PDNAPFPKDDSGLR---- 607

OAG18414.1_Laccase_Asco LGVQFLERSSEIKAKDGFA---EMKRTCKNWQTYHRIF---HPNGILFPG-DSGL----- 603

OAL06359.1_Stago_tyrosinase ------------------------------------------------------------ 361

CAC82195.1_Tyrosinase_Asco ------------------ERQEWHNLGKLL---PVKETADIIRQHLCWKVVGV-NNSILF 576

GFP57945.1_PPO_Asco ---------------------------------N-RNAVSAFSSDVEVEILTR-SGQSIP 482

EAA35587.2_Tyrosinase_Asco -----FRGEDE----GYGEDGDWVGQGEVLRSLDPKDVEPFLRRNLVVNIVKM-DGKVVM 661

OAT05486.1_PPO_Asco ------------------------DSKMILD-LEDATILPLLEKHLEWRVQDL-TGKVVD 577

PBP18673.1_Tyrosinase_Asco --------------------------EMNRATHDASATTDYLKKSLNWSVQKF-DGTVVP 577

XP_031001670.1_PPO_Asco --------------------------G--VEAGDVTAVVEYLKENLRWSVVKN-SDG-SV 540

RMI99061.1_Bilirubin_Asco ARANPYGDVDA---VEDALKEYWAKK-------------NAAKKDT---------ESRSI 608

OAL02734.1_Stago_bilirubin AQQQPYSEQAQ---VEAALAAAWADG-------------SAQKRSVGEECGGQ--SSGPI 618

XP_018030874.1_Para_bilirubin AREQPYSELAE---VEAALDVAWADG-------------TAQKRS------------GVI 605

RYN74507.1_Bilirubin_Asco EALEAYTGPEK---LEDALVDYWANG-------------GKGPSTLVTSTRPASSSSS-- 573

EAA27114.3_Bilirubin_Asco AGFRPYDKVEE---VESVLDQYWETK-------------TKAGSTATQVV-PTTISAGVF 587

KAF1967560.1_Ascorbate_oxid_Asco VVWDEFDGEKDKDGH--------------------------------------------- 609

BAA24288.1_Ascorbate_oxid_Asco ------------------------------------------------------------ 551

KYK60778.1_Ascorbate_oxid_Asco TAMHEFDDAKPESCPGQNAASQRLDD---------AGQDAAGQS----------PG---- 621

GAQ36284.1_Laccase_Asco ------------------------------------------------------------ 620

CAA70509.1_MCO_Asco -----YLDL-----KGENVQVKRLPT----------GFTTKGIVALVFSCVAAFLGLFSF 574

PTB67553.1_MCO_Asco -----FLDL-----TGENKAPGTIPG----------GFTPRGIVALVFSCITGVLGVLVV 568

KJK64339.1_MCO_Asco -----LLDL-----TGQNKQLAWLPA----------GFTARGIVALVFSCISAVLGLGFI 586

AAA33591.1_Laccase_Asco ------SGV-----KAREVKMKW------------------------------------- 619

OAG18414.1_Laccase_Asco ------------------------------------------------------------ 603

OAL06359.1_Stago_tyrosinase ------------------------------------------------------------ 361

CAC82195.1_Tyrosinase_Asco D-------SEQPMRGDPATWRSLDVTAAYSDIHYPVDRNYKYIDRGLPAYHNYL------ 623

GFP57945.1_PPO_Asco L-------ESVPS---------LAIDLESANVTM-----P-SAIDQLPKYLTRSKHRAQA 520

EAA35587.2_Tyrosinase_Asco H-------GSYVEG--------LGIHVVSSRVRA-----A-RVEEELPAWGQAESGFDVV 700

OAT05486.1_PPO_Asco A-------GELIGS-STGHSCGLEITIAERDVTP-LTGDE-KERDHFPILGEWKMFKDIT 627

PBP18673.1_Tyrosinase_Asco V-------EDIPS---------LTVTVQDELVTD-----S-GDITKLPTYGKSTTHPEVT 615

XP_031001670.1_PPO_Asco V-------GDVEG---------LEVEVADEIVEP-----A-NDIFMFPSYGDRTVHPEIT 578

RMI99061.1_Bilirubin_Asco P-----------------RFRRFQV----------------------------------- 616

OAL02734.1_Stago_bilirubin S-----------------RVRRFVV----------------------------------- 626

XP_018030874.1_Para_bilirubin P-----------------RFRRFIM----------------------------------- 613

RYN74507.1_Bilirubin_Asco --QATASSTALPTTTSPPSVARVTSSSAAITS--A---------P-----KATS------ 609

EAA27114.3_Bilirubin_Asco TTSVKASTTSFKT------------STASSSS--S---------T-----RTSS------ 613

KAF1967560.1_Ascorbate_oxid_Asco ------------------------------------------------------------ 609

BAA24288.1_Ascorbate_oxid_Asco ------------------------------------------------------------ 551

KYK60778.1_Ascorbate_oxid_Asco -----------KGPGQNAASQRLDDAGQDAAGQSP---------GKGPGQNAAS------ 655

GAQ36284.1_Laccase_Asco ------------------------------------------------------------ 620

CAA70509.1_MCO_Asco S--FYGMND--IAHVEDKVARDLDIDLEAENE-DE---------EEAVVLNQNS------ 614

PTB67553.1_MCO_Asco A--WYGLST--PLELVPAAVARVVETSEVADD-DD---------GHH--SSSSA------ 606

KJK64339.1_MCO_Asco A--FYGMSG--LPSSE-----RTQEQGEGENG-PD---------GRI--APSES------ 619

AAA33591.1_Laccase_Asco ------------------------------------------------------------ 619

OAG18414.1_Laccase_Asco ------------------------------------------------------------ 603

OAL06359.1_Stago_tyrosinase ------------------------ 361

CAC82195.1_Tyrosinase_Asco -----------PIHLSPT------ 630

GFP57945.1_PPO_Asco AQKGQRFQPPPPPPPPPSQ----- 539

EAA35587.2_Tyrosinase_Asco ------------------------ 700

OAT05486.1_PPO_Asco QKEKKKKQCKPKYKV--------- 642

PBP18673.1_Tyrosinase_Asco RGRAGGYSGVA------------- 626

XP_031001670.1_PPO_Asco TGLY-------------------- 582

RMI99061.1_Bilirubin_Asco ------------------------ 616

OAL02734.1_Stago_bilirubin ------------------------ 626

XP_018030874.1_Para_bilirubin ------------------------ 613

RYN74507.1_Bilirubin_Asco -SSTKKDDKKTSTS--STRRR--- 627

EAA27114.3_Bilirubin_Asco -TSTKRKD---------------- 620

KAF1967560.1_Ascorbate_oxid_Asco ------------------------ 609

BAA24288.1_Ascorbate_oxid_Asco ------------------------ 551

KYK60778.1_Ascorbate_oxid_Asco -QSPGKGPGQNAAS---QRLDDAG 675

GAQ36284.1_Laccase_Asco ------------------------ 620

CAA70509.1_MCO_Asco -SSSDSNSKPH------------- 624

PTB67553.1_MCO_Asco -GAAGNGSGAAAAAAGAATLRS-- 627

KJK64339.1_MCO_Asco -GAL----------------RE-- 624

AAA33591.1_Laccase_Asco ------------------------ 619

OAG18414.1_Laccase_Asco ------------------------ 603

**
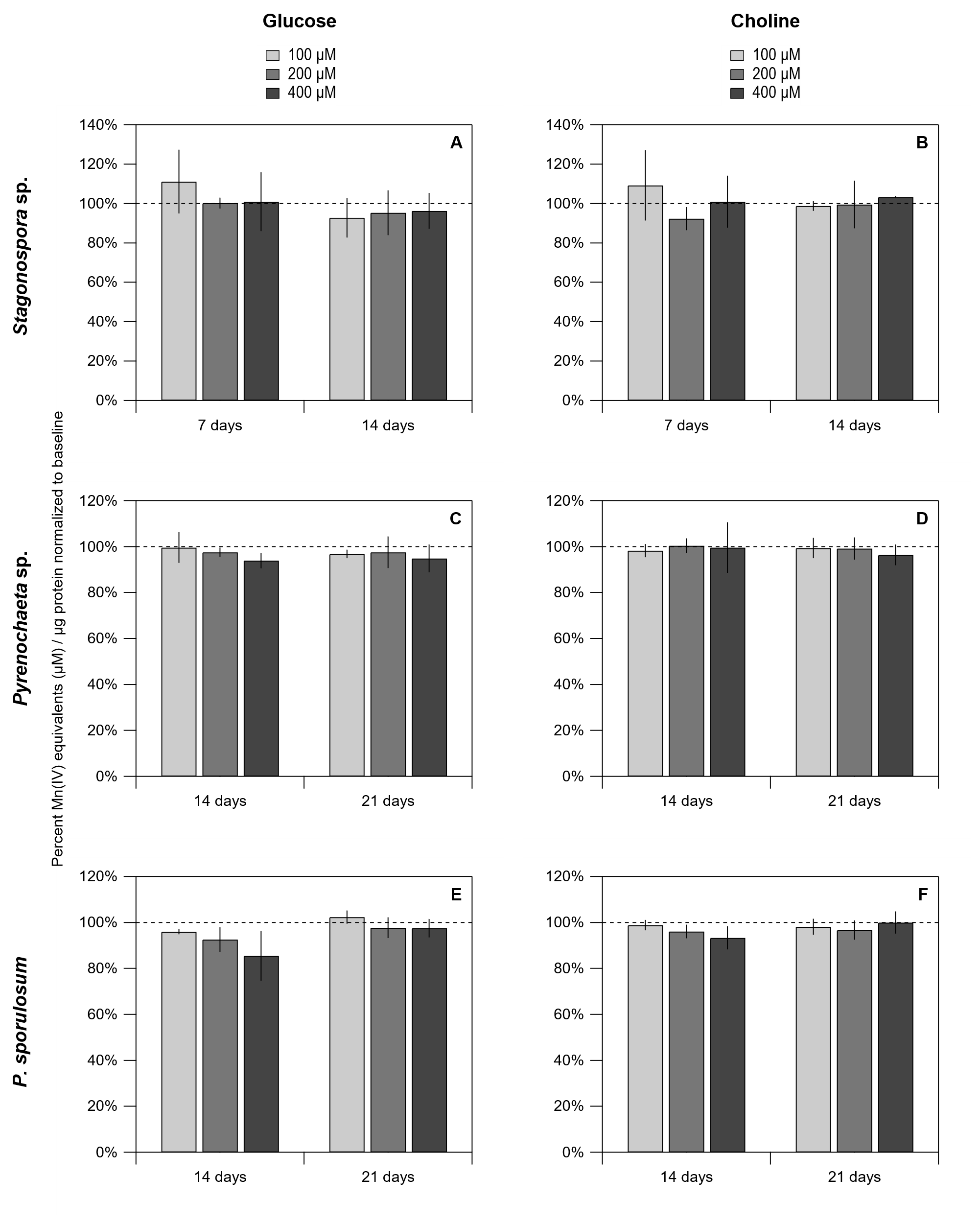
**

**Figure S4. Mn(II) oxidation was uninhibited by D-glucose and choline in the secretomes of all 3 fungi.** Mn(II) oxidation in cell-free secretomes of (A-B) *Stagonospora* sp., (C-D) *Pyrenochaeta* sp., and (E-F) *P. sporulosum* in the presence of 100 µM, 200 µM, or 400 µM D-glucose (left) or choline chloride (right). All samples were supplemented with 350 µM Mn(II) and incubated for 1 hour before Mn oxide quantification. All data are normalized to a matrix control (deionized H_2_O) with 0 µM reagent. Error bars represent +/- 1 standard deviation (N=3 for *Stagonospora* sp. and *Pyrenochaeta* sp.; N=4 for *P. sporulosum*).

**
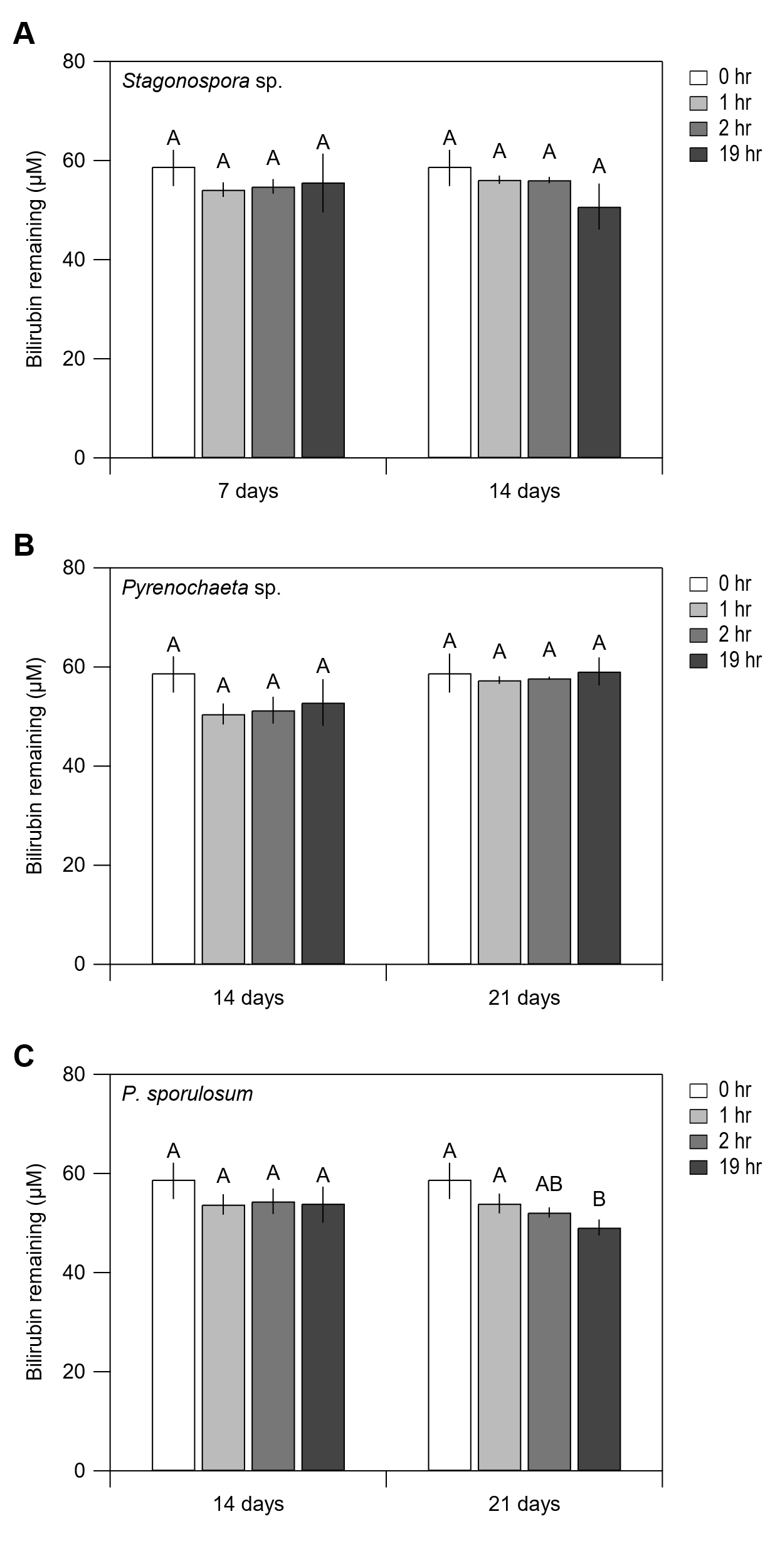
**

**Figure S5.** **Fungal secretomes did not significantly oxidize unconjugated bilirubin, except the 21-day-old *P. sporulosum* secretome.** Cell-free secretomes of (A) *Stagonospora* sp., (B) *Pyrenochaeta* sp., and (C) *P. sporulosum* were incubated with 60 μM bilirubin in 8.8% DMSO, buffered by 20 mM HEPES pH 8.0, for 1, 2, or 19 hours. Bilirubin oxidation was measured via absorbance at 450 nm. *Stagonospora* sp. experiments were conducted with 1.0 μg fungal protein, while all others used 0.5 μg fungal protein. Data were analyzed with a one-way ANOVA followed by a Tukey-Kramer post-hoc test; within each organism, letters indicate statistically different groups. Error bars represent +/- 1 standard deviation (N=3 for *Stagonospora* sp. and *Pyrenochaeta* sp.; N=4 for *P. sporulosum*).

**Figure S6.**  **Acetate in *Pyrenochaeta* sp. liquid cultures was depleted after 14d of growth.** Cell-free secretomes were analyzed with a 1D 600 Mhz nuclear magnetic resonance (NMR) spectrometer, and peaks were mapped to a database of common metabolites and amino acids. Plotted are acetate (the primary carbon source in AY medium) and uracil (the RNA nucleobase). RNA could be present in the cultures as a component of initial growth medium ingredients and through production by fungal biomass during growth. Data at 0 days represent AY-Mn medium controls. Error bars represent +/- 1 standard deviation over 4 biological replicates at each time point.
